# Spatio-Temporal Dynamics of M_1_ and M_2_ Macrophages in a Multiphase Model of Tumor Growth

**DOI:** 10.1007/s11538-025-01466-6

**Published:** 2025-06-04

**Authors:** Ioannis Lampropoulos, Panayotis G. Kevrekidis, Christos E. Zois, Helen Byrne, Michail Kavousanakis

**Affiliations:** 1 School of Chemical Engineering, National Technical University of Athens, Iroon Polytechneiou 9, Zografou, Athens, 15780 Greece; 2 Department of Mathematics and Statistics and Department of Physics, University of Massachusetts Amherst, Amherst, 01003 Massachusetts United States of America; 3 Department of Oncology, University of Oxford, Old Road Campus Research Building, Oxford, OX3 7DQ England; 4 Department of Radiotherapy and Oncology, Democritus University of Thrace, Dimokritou 7A, Komotini, 68100 Greece; 5 Mathematical Institute, University of Oxford, Wellington Square, Oxford, OX1 2JD England; 6 Ludwig Institute for Cancer Research, University of Oxford, Wellington Square, Oxford, OX1 2JD England

**Keywords:** Multiphase model, Immunotherapy

## Abstract

This study investigates the complex dynamics of vascular tumors and their interplay with macrophages, key agents of the innate immune response. We model the tumor microenvironment as a multiphase fluid, with each cellular population treated as a distinct, non-mixing phase. The framework also incorporates diffusible species that are critical for processes such as nutrient transport, angiogenesis, chemotaxis, and macrophage activation. A central contribution of this work is the explicit modeling of macrophage infiltration and polarization within the tumor microenvironment. The model captures the divergent roles of $\hbox {M}_1$ (anti-tumor) and $\hbox {M}_2$ (pro-tumor) macrophages and their influence on tumor aggressiveness and progression. Through numerical simulations, we demonstrate the emergence of both spatial and phenotypic heterogeneity in the macrophage population, including their peripheral localization and limited core infiltration -patterns consistent with experimental observations. Furthermore, this is the first multiphase model to incorporate the effects of TGF-$\beta$-targeting immunotherapy using vactosertib. Our simulations demonstrate that treatment initially enhances the presence of anti-tumor macrophages, followed by a relapse period where tumor dynamics returns to pre-treatment trends. Model parameters are grounded in experimental data and clinically relevant dosage protocols.

**Supplementary Information:**

The online version contains supplementary material available at 10.1007/s11538-025-01466-6.

## Introduction

Understanding interactions between cancer cells and the immune system is a challenging research topic. Cancer can manipulate a host’s immune system to avoid eradication and even cooperate with it (Hiam-Galvez et al. 2021; Gonzalez et al. 2018). In fact, cancer cells can remodel the immune system, causing cells to differentiate towards specific cell lineages which promote tumor growth (Canadian Cancer Society 2024; Hiam-Galvez et al. 2021; Gonzalez et al. 2018). This ability contributes crucially to chronic inflammation, which in turn helps cancer growth and dissemination (Gonzalez et al. 2018).

As highlighted in Dunn et al.’s seminal work (Dunn et al. 2004), the immune system and cancer cells engage in continuous, dynamic interactions that shape tumor development. This complex process, termed *immunoediting*, is divided into three stages, often referred to as the three "E"s. The first stage, *Elimination*, occurs when the immune system successfully detects and destroys cancer cells. The second stage, *Equilibrium*, represents the phase in which the immune system manages to control tumor growth without completely eradicating it. Finally, in the *Escape* phase, some tumor cells adapt by developing mechanisms to evade immune detection and proliferate unchecked.

Among the first immune cells to mobilize after tumorigenesis are macrophages (Hiam-Galvez et al. 2021), products of monocyte differentiation (Pittet et al. 2014; Perdiguero and Geissmann 2016; Ginhoux and Guilliams 2016). Monocytes are found in the circulatory system and, as they extravasate into damaged tissues, they differentiate into macrophages (Strell and Entschladen 2008). During tumor growth, macrophages play pivotal yet contradicting roles. The significant variance in their functions stems from the dynamic nature of their phenotype. Macrophages can adopt a spectrum of phenotypes in response to their microenvironment (Bied et al. 2023; Gao et al. 2022; Ross et al. 2021). Two prominent phenotypes are classically activated macrophages, $\hbox {M}_1$, and alternatively activated macrophages, $\hbox {M}_2$. The former exhibit pro-inflammatory characteristics and inhibit tumor progression, while the latter tend to be anti-inflammatory and promote tumor progression (Mosser and Edwards 2008; Gao et al. 2022). Consequently, lower ratios of $\hbox {M}_1$ to $\hbox {M}_2$ are associated with poorer prognosis for cancer patients (Vito et al. 2020).

Numerous molecules are involved in shaping macrophage phenotype, and thereby, behavior. While considering all signaling molecules is beyond the scope of this study, it is important to examine the roles of certain key factors. Consider for example colony stimulating factor-1 (CSF-1), which is secreted by both cancer cells and tumor associated macrophages (TAMs). CSF-1 binds to CSF1R receptors which are highly expressed on the surface of macrophages and biases their movement towards the tumor. Effectively, CSF-1 can be viewed as a macrophage chemoattractant. At the same time, in response to environmental cues such as hypoxia, TAMs express cytokines such as the epidermal growth factor (EGF). Cancer cells which express EGF receptors, then migrate towards the TAMs, completing a paracrine loop, which facilitates cancer cell movement and may contribute to migration and metastasis (Leung et al. 2017; Wyckoff et al. 2004; Goswami et al. 2005).

TAMs contribute to tumor metastasis, as they attract cancer cells towards the vasculature via EGF-mediated paracrine signaling (Leung et al. 2017; Wyckoff et al. 2004; Goswami et al. 2005; Arwert et al. 2018). C-X-C motif chemokine 12 (CXCL12), also known as stromal cell-derived factor 1 (SDF-1), stimulates migration in most leukocytes (Janssens et al. 2018), including TAMs. As a result, CXCL12 secreted by perivascular fibroblasts coupled with EGF-mediated paracrine signaling, drives migration of $\hbox {M}_2$ macrophages and tumor cells towards the vasculature to promote metastasis (Arwert et al. 2018).

Macrophage behavior within a diseased tissue depends on its phenotype. Several factors determine a macrophage’s phenotype, including molecular signals and environmental conditions (Italiani et al. 2016). For example, transforming growth factor beta (TGF-$\beta$) drives macrophage polarization towards the $\hbox {M}_2$ (pro-tumor) phenotype. As such, TGF-$\beta$ represents a natural target for immunotherapy (Arwert et al. 2018; Zhang et al. 2016).

Another important mediator is vascular endothelial growth factor (VEGF). VEGF is a diffusible cytokine, secreted by cancer cells under hypoxia. It stimulates mature endothelial cells to shed their protective pericyte layer and to proliferate. VEGF also maintains vascular networks in an immature state, where they lack proper structure and are prone to leaks and occlusion (Darland et al. 1999; Gee et al. 2003). Like tumor cells, TAMs secrete VEGF under hypoxia. This stimulates angiogenesis, providing the tumor with increased access to nutrients and facilitating metastasis (Wu et al. 2010).

Numerous computational studies have attempted to unravel the mechanisms underlying macrophage involvement in cancer growth. Initial modeling efforts focused on their anti-cancer pro-inflammatory behavior (Owen and Sherratt 1997, 1998, 1999; Kelly et al. 2002), and their potential to serve as carriers for therapeutic agents (Owen et al. 2004; Webb et al. 2007; Leonard et al. 2016; Boemo and Byrne 2019). More recent, models have focused on the phenotypic plasticity of macrophages and their ability to perform different functions depending on their microenvironment. Several ordinary differential equation (ODE) models have been developed to investigate macrophage polarization (Louzoun et al. 2014; den Breems and Eftimie 2016; Eftimie 2020; Eftimie and Barelle 2021). The role of macrophages in diseased sites has also been studied using multi-scale models (Mahlbacher et al. 2018; Leonard et al. 2020; Suveges et al. 2022).

Regarding macrophage interactions within their micro-environment, Knútsdóttir et al. (2014) studied various paracrine and autocrine loops, including the CSF-1/EGF loop discussed earlier, in order to understand how macrophages enable metastasis in breast cancer. Norton et al. (2018) developed an agent-based model demonstrating that macrophages promote and facilitate cell migration and metastasis. Their findings suggested that invasive tumor cells rely on recruited macrophages to sustain their metastatic potential. Finally, Bull and Byrne (2023) utilized an agent-based model to validate a novel weighted pair correlation function to investigate the spatio-temporal evolution of interactions between cancer cells and macrophages, study macrophage phenotype switching, paracrine/autocrine signaling and metastasis. In their study they showed how varying the rate of macrophage extravasation and macrophage chemotactic sensitivity to CSF-1 affect the ability (or inability) of macrophages to eliminate a tumor.

Tumor growth modeling that treats the tissue as a continuum has been the focus of numerous scientific studies, even when macrophages were not the primary focus. These works have significantly influenced the literature and shaped the direction of scientific research (Ward and King 1997; Byrne and Preziosi 2003; Hubbard and Byrne 2013; Breward et al. 2003, 2002). The rationale for employing a continuum level approach stems from its ability to effectively capture the macroscopic dynamics of tumor growth,and immune interactions across spatial and temporal scales relevant to clinical observations.

In this study, we present a multiphase model in a two-dimensional domain. Our model focuses on the complex behavior of macrophages within tumors. Naturally, the trade-off is that the present model does not provide the detailed local information that, e.g., the agent-based approach of Bull and Byrne (2023) allows. It accounts for macrophage recruitment through the vasculature and the paracrine signaling that induces monocyte extravasation. Furthermore, the model tracks phenotype switching of macrophages from a pro-inflammatory to a pro-tumor state. In addition, the model investigates the role of cancer cells’ secretions and environmental factors -such as hypoxia- on alternative activation. We also investigate the impact of TAMs on tumor invasion by monitoring changes in the tumor’s growth rate in the presence of macrophages. Another feature of our model is the representation of hypoxic niches within the tumor and the simulation of TAM concentrations in these hypoxic regions.

The aim of immunotherapy is to enhance the immune system’s ability to recognize and eliminate cancer cells. Immunotherapeutic strategies include checkpoint inhibitors, CAR-T cell therapy, and cancer vaccines, which are designed to reinvigorate the immune system and re-establish immune surveillance against cancer (Alard et al. 2020). We use our model to investigate the effect of immunotherapy that inhibits TGF-$\beta$/TGF-$\beta$R signaling on classically activated macrophages, motivated by the compound vactosertib. This drug binds to TGF-$\beta$ receptors on macrophage surfaces, preventing the binding of TGF-$\beta$ and, consequently, alternative activation. This mechanism effectively “freezes" the macrophage phenotype in a pro-inflammatory state (Pubchem 2024; Jung et al. 2020). Vactosertib is currently undergoing clinical trials and has shown promise as an immunotherapeutic agent (Samsung Medical Center (Responsible Party (2024); Case Comprehensive Cancer Center (Responsible Party) (2024a, 2024b); Yonsei University (Responsible Party) (2024)). This therapy reprograms macrophages by blocking the downstream signaling pathways responsible for activating the pro-tumor phenotype (Shojaee et al. 2022; Gao et al. 2022; Mantovani et al. 2022; Duan and Luo 2021; Zhao et al. 2022), rather than eliminating them or targeting other signaling pathways such as CSF-1/CSF-1R (Shojaee et al. 2022; Shields IV et al. 2020; Pyonteck et al. 2013; Ries et al. 2014). In our study, we simulate vactosertib’s effect on macrophage behavior, and the subsequent effect on tumor growth. We monitor the immune response that is achieved with the help of vactosertib and compare it to the therapy-free scenario. Lastly, we consider the impact certain kinetic parameters have on the drug’s therapeutic efficacy.

Our paper is structured as follows: Section 2 presents the model developed for an untreated tumor scenario. Section 3 describes computational methods employed, including technical details about the numerical solution of the developed system of equations. Section 4 presents our findings, including the influence of macrophages on tumor growth, and the spatio-temporal distributions of TAMs within the tumor microenvironemnt. Section 5 introduces the model for a tumor treated with immunotherapy. Section 6 explores the impact of immunotherapy based on our findings. Finally, section 7 provides concluding remarks on the presented results and outlines potential directions for future research.

## Therapy free model

We introduce a two-dimensional, multiphase model consisting of six interacting phases: healthy cells, cancerous cells and macrophages ($\hbox {M}_1$ and $\hbox {M}_2$), along with vascular and interstitial fluid phases. Each phase is modeled as a viscous fluid and characterized by the spatio-temporal evolution of its normalized concentration, denoted as $\theta _i$, with *i* representing each fluid phase:

- Healthy cells, $$\theta _h$$.
- Cancer cells, $$\theta _c$$.
- $$\hbox {M}_1$$ macrophages, $$\theta _{M_1}$$.
- $$\hbox {M}_2$$ macrophages, $$\theta _{M_2}$$.
- Vasculature, $$\theta _v$$.
- Interstitial fluid, $$\theta _{int}$$.

We associate with each phase a velocity vector, $\vec {u}=\left( u_i,v_i \right)$, and a pressure term, $p_i$, ($i=h$, *c*, $M_1$, $M_2$, *v*, *int*).

In addition to the fluid phases, the model accounts for several diffusible chemical species:

- Oxygen, *c*: a representative for nutrients in the system.
- VEGF, *g*: stimulates angiogenesis.
- CSF-1, *a*: mediates macrophage extravasation.
- CXCL12, *b*: acts as a chemoattractant for $\hbox {M}_2$ macrophages.
- EGF, *l*: attracts cancer cells, facilitating tumor expansion and metastasis.
- TGF-$\beta$, *f*: polarizes macrophages from $\hbox {M}_1$ to $\hbox {M}_2$.

We assume that the chemical species do not contribute to the system’s volume, as they are soluble. Oxygen is replenished via the vascular network, while the remaining diffusible species are expressed by specific cellular populations. Table 1 summarizes the variables defined in our system.

**Fig. 1:**
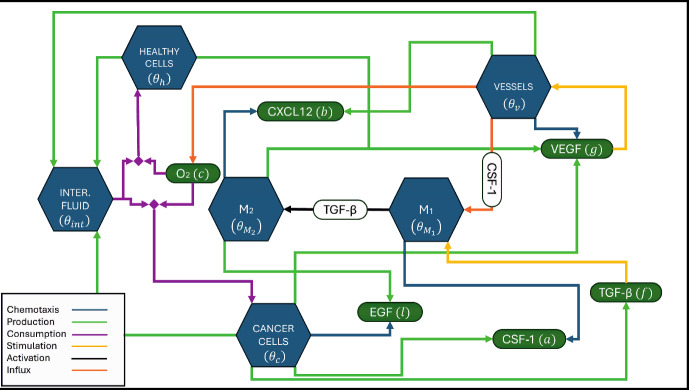
Model schematic. In the interactions presented, chemotaxis (and subsequent binding of molecules to the relevant receptors) is represented by blue arrows, production/secretion is illustrated by green arrows, consumption is shown as purple arrows, and influx through vasculature/extravasation is depicted by orange arrows. The rounded rectangles represent chemical species: green shapes indicate diffusible molecules that are produced or consumed during biological processes, while white shapes denote chemical species that trigger biological processes (Color figure online)

A schematic of the model, depicting the interactions between the different cellular phases and chemical species is presented in Fig. 1.

**Table 1:** List of the model’s variables

Variable name	Description	Variable name	Description
$\theta _h$	Healthy cells concentration	*g*	VEGF concentration
$\theta _c$	Cancer cells concentration	*a*	CSF-1 concentration
$\theta _v$	Vessels concentration	*b*	CXCL12 concentration
$\theta _{int}$	Interstitial fluid concentration	*l*	EGF concentration
$\theta _{M_1}$	$\hbox {M}_1$ concentration	*f*	TGF-$\beta$ concentration
$\theta _{M_2}$	$\hbox {M}_2$ concentration	*d*	Drug concentration
$\theta _{M_{1_p}}$	Drug-bound $\hbox {M}_1$ concentration	$\vec {u_i}$	Fluid phase, *i*, velocity
*c*	Oxygen concentration	$p_i$	Fluid phase, *i*, pressure

### Mass balance equations for cellular phases

**Fig. 2:**
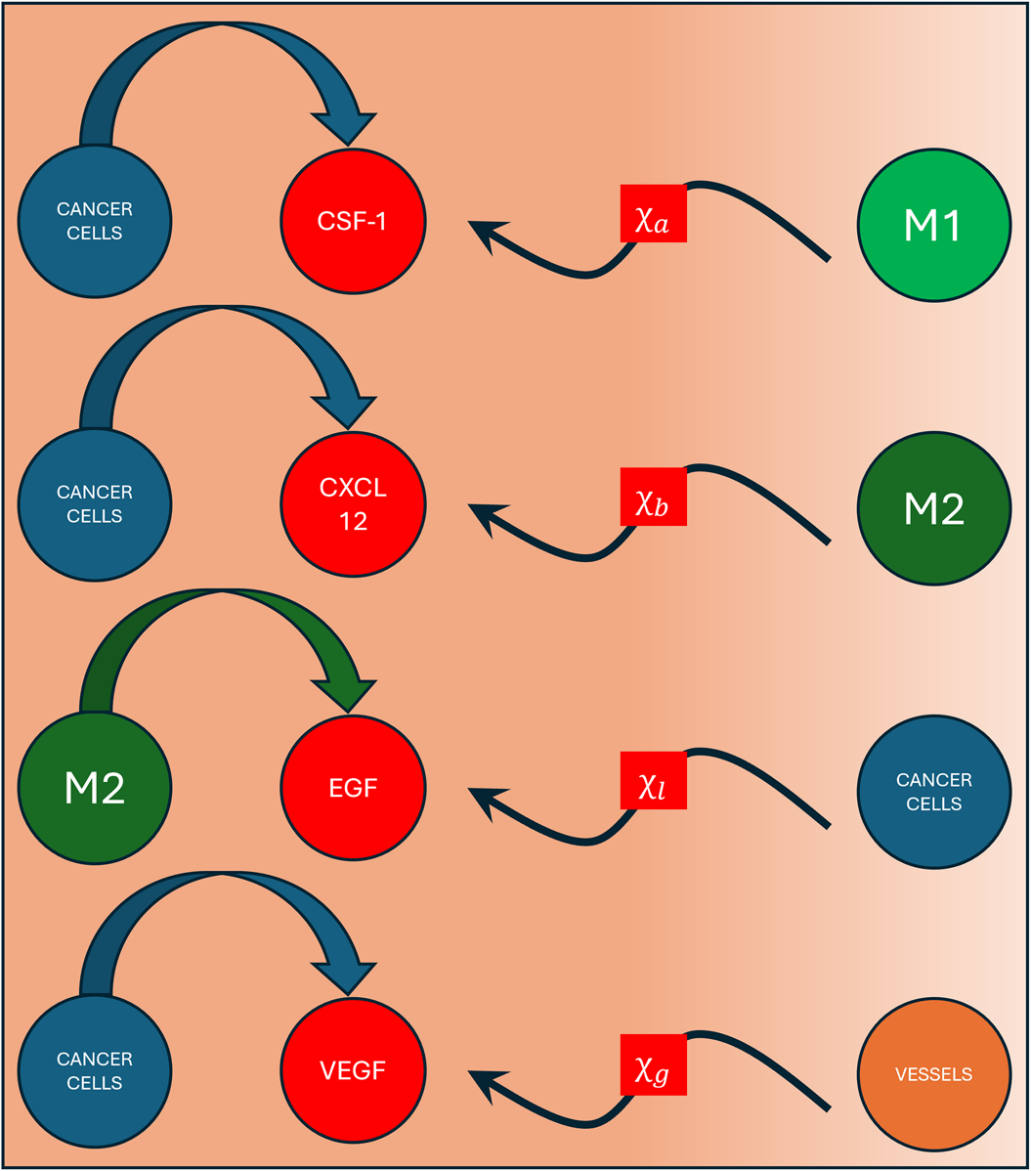
A summary of the chemokines included in our model, along with the cells that secrete them and the cells which are chemosensitive to them

It is commonly assumed that, at a macroscopic level, the density of living tissue is uniform (Liu et al. 2022). As a result, the mass balance equations for the cellular phases are formulated in the following general form:

1 $$\begin{aligned} \frac{\partial \theta _i}{\partial t} + \nabla \cdot \left( \vec {u}_i \theta _i \right) + \chi _{J} \nabla \cdot \left( \theta _i \nabla J \right) = q_i, \end{aligned}$$

where $i=h,c,M_1,M_2,v,int$ represents various phases, and *J* denotes the concentration of a chemical species, which acts as a chemoattractant for phase, *i*. In our model, there is at most one chemoattractant per cellular phase. Those pairings are listed in Table 2. The terms associated with mass transport appear on the left side of Eq. (1). In particular, $\nabla \cdot \left( \vec {u}_i \theta _i \right)$ represents mass transport through convection, while $\chi _{J} \nabla \cdot \left( \theta _i \nabla J \right)$ represents mass transfer of cellular phase, *i*, due to chemotaxis of spatial gradients of cytokine, *J*. The parameter $\chi _{J}$ denotes the strength of chemoattraction between cellular phase, *i*, and chemoattractant, *J*. Finally, the term $q_i$ denotes the net production term for phase *i*. Not every chemokine attracts every cell type. Figure 2 illustrates the production and targets of our system’s chemoattractants, offering a summary of the above.

**Table 2:** Chemokine sensitive cells and their designated chemokine

Cell/Fluid phase	Chemokine/Chemical species	Source
Cancer cells $\left( c\right)$	EGF $\left( l\right)$	(Harney et al. 2015)
Vasculature $\left( v\right)$	VEGF $\left( g\right)$	(Darland et al. 1999; Gee et al. 2003)
Pro-inflammatory macrophages $\left( M_1\right)$	CSF-1 $\left( a\right)$	(Wang et al. 2021)
Pro-tumor macrophages $\left( M_2\right)$	CXCL12 $\left( b\right)$	(Arwert et al. 2018; Janssens et al. 2018)

Boundary conditions for the mass balance equations (Eq. (1)) are specified on inflow segments of the tissue boundary ($\partial \Omega$). Inflow segments are defined as sections of $\partial \Omega$ where $\vec {u}_i \cdot \vec {n} < 0$, with $\vec {n}$ denoting the unit normal vector to the boundary. On inflow boundary segments, denoted $\partial \Omega ^{inflow}$, we impose Dirichlet boundary conditions:

2 $$\begin{aligned} \theta _i = \theta _i^{\infty }, i=h,c,v,int,M_1,M_2, \end{aligned}$$

where $\theta _i^{\infty }=\theta _{i_{init}}$, the concentration of species *i* at time $t=0$. Homogeneous Dirichlet boundary conditions are imposed to simulate the state of a healthy tissue outside the domain of interest, $\Omega$.

### Production terms for mass balance equations

We now present the production terms, $q_i$ $\left( i=h,c,v,int,M_1,M_2\right)$, for the volume occupying phases.

#### Healthy cells

We assume the production term for the healthy tissue surrounding the tumor can be written as:

3 $$\begin{aligned} q_h = k_{m,h} \theta _h \theta _{int} \frac{c}{c_p+c} -k_{d,h} \theta _h \frac{c_{c_1} + c}{c_{c_2} + c}. \end{aligned}$$

In Eq. (3), the first term simulates cellular proliferation and the second term represents cell death. We assume that healthy cells proliferate at a rate which is an increasing saturating function of the oxygen concentration, *c*, with a maximum value $k_{m,h}$. The parameter $c_p$ is the oxygen concentration at which the proliferation rate is half maximal. The proliferation rate is proportional to the interstitial fluid concentration, $\theta _{int}$ (the interstitial fluid supplies all essential components required for cellular proliferation Hubbard and Byrne 2013; Breward et al. 2003) and the healthy cells concentration, $\theta _h$. The second term models cell death due to apoptosis and nutrient scarcity. We assume that it is a decreasing function of oxygen concentration, *c*, and, hence, that the parameters $c_{c_1},c_{c_2}$ satisfy $c_{c_1}>c_{c_2}$. Further, $k_{d,h}$ represents the basal rate of healthy cells death, attained in the limit $c\rightarrow +\infty$.

#### Cancer cells

The cancer cells proliferate and die, like the healthy cells. Additional terms are included to account for the boost in cancer cell proliferation caused by the presence of $\hbox {M}_2$ macrophages and the increase in cancer cell death caused by $\hbox {M}_1$ macrophages killing their targets:

4 $$\begin{aligned} q_c&= \left( k_{m,c} + k_{c,M_2} \frac{\theta _{M_2}}{\theta _M+\theta _{M_2}} \right) \theta _{c} \theta _{int} \frac{c}{c_p+c} - k_{d,c} \theta _{c} \frac{c_{c_1} + c}{c_{c_2} + c}\nonumber \\&\quad - k_{c,M_1} \theta _c \frac{\theta _{M_1}}{\theta _M+\theta _{M_1}}. \end{aligned}$$

In Eq. (4), $k_{m,c}$ denotes the maximum proliferation rate of cancer cells, and $k_{d,c}$ is the rate at which cancer cells die. The cancer cell growth due to pro-tumor macrophage ($\hbox {M}_2$) presence is included in the cancer cell proliferation term. $k_{c,M_2}$ is the maximum increase in cancer cell proliferation due to the presense of $\hbox {M}_2$ macrophages. $\theta _M$ is the concentration of macrophages where the rate of interaction between them and the cancer cells becomes half maximal. On the other hand, the killing of cancer cells caused by $\hbox {M}_1$ macrophages is presented as a separate term. There, $k_{c,M_1}$ represents the maximum rate for the interactions between cancer cells and $\hbox {M}_1$ macrophages, resulting to the death of the former. For simplicity, it is assumed that $\theta _M$ is common for interactions between cancer cells and both $\hbox {M}_1$ and $\hbox {M}_2$ macrophages. It is assumed that $c_p$, $c_{c_1}$, and $c_{c_2}$ parameter values are identical for both healthy somatic and cancerous cells. Given the characteristics of malignant tumors consisting of rapidly proliferating and highly resilient cells, $k_{m,c} > k_{m,h}$ and $k_{d,c} < k_{d,h}$.

#### Vasculature

The net production term for the vascular phase is given by:

5 $$\begin{aligned} q_v = k_{ang} \theta _{v} g \frac{\theta _{int}}{\epsilon + \theta _{int}} - k_{occ} \theta _{v} \mathcal {H} \left( p_{cell} - p_{crit}, h \right) . \end{aligned}$$

The first term in Eq. (5) describes angiogenesis, the process by which new blood vessels form from existing vessels (Darland et al. 1999). We suppose that this rate is an increasing, saturating function of $\theta _{int}$ and proportional to the local concentration of VEGF, *g*. The interstitial fluid provides the material needed for endothelial cell proliferation. The parameter $k_{ang}$, represents this phenomenon’s maximum rate, for a given VEGF concentration, *g*.

Tumor vessels are typically immature and prone to collapse (Darland et al. 1999). We assume that blood vessels become occluded when the pressure exerted on them by the surrounding cellular phases exceeds the threshold value $p_{crit}$. The value of $p_{crit}$ represents the maximum pressure that the vessels can withstand. $p_{cell}$ denotes the total pressure exerted by the cellular phases on the vessels:

6 $$\begin{aligned} p_{cell}=\frac{1}{\sum _{i}^{6}\theta _i} \left( \theta _h p_h+\theta _c p_c+ \theta _{M_1} p_{M_1}+\theta _{M_2} p_{M_2}\right) . \end{aligned}$$

In Eq. (6), $p_h$ and $p_c$ denote the pressures exerted by the healthy and cancer cells and, similarly, $p_{M_1}$ and $p_{M_2}$ are the pressures exerted by the $\hbox {M}_1$ and $\hbox {M}_2$ macrophages. $\sum _{i}^{6}\theta _i$ denotes the sum of concentrations of all cellular phases considered in the model, including the macrophage species. Finally, in Eq. (5), $\mathcal {H}$ is a smooth approximation to the Heaviside function:

7 $$\begin{aligned} \mathcal {H} \left( y, h \right) = \frac{1}{2} \left[ 1+ \tanh \left( \frac{y}{h} \right) \right] , \end{aligned}$$

where the parameter *h* controls the smoothness $\left( h<<1\right)$.

#### Macrophages $(\hbox {M}_1$ and $\hbox {M}_2)$

The production term for pro-inflammatory $\hbox {M}_1$ macrophages is formulated as follows:

8 $$\begin{aligned} q_{M_1} = k_{ext,M_1} \theta _v \frac{a}{a_p+a} \underbrace{-k_{d,M_1} \theta _{M_1} - k_{aa} \theta _{M_1} \frac{f}{f_p+f}}_{\overline{q_{M_1}}}. \end{aligned}$$

The first term represents the supply of macrophages from the vascular network. The macrophage extravasation rate is an increasing, saturating function of CSF-1’s concentration, *a*, with $k_{ext,M_1}$ representing its maximum value. $a_p$ is the CSF-1 concentration at which extravasation rate is half maximal. While there are reports of TAM proliferation in the tumor micro-environment, it is neglected in this study, motivated by experimental studies which show that most of the macrophages within the tumor microenvironment originate from differentiation of circulating monocytes (Pittet et al. 2014; Shojaee et al. 2022; Mantovani et al. 2017).

We group the remaining two terms in $q_{M_1}$ into a single sink term $\overline{q_{M_1}}$ in order to distinguish them from the recruitment term which introduces mass to the system. The two sink terms represent: (i) macrophage death at a rate $k_{d,M_1}$, and (ii) alternative activation, the process by which $\hbox {M}_1$ macrophages transition to a pro-tumor $\hbox {M}_2$ phenotype. We assume that this process is an increasing, saturating function of TGF-$\beta$, *f*, with maximum rate $k_{aa}$, and $f_p$ being the TGF-$\beta$ concentration at which the transition rate is half maximal.

The production term for $\hbox {M}_2$ macrophages is similar to that of $\hbox {M}_1$ macrophages:

9 $$\begin{aligned} q_{M_2} = k_{aa} \theta _{M_1} \frac{f}{f_p+f} - k_{d,M_2} \theta _{M_2}. \end{aligned}$$

In Eq. (9), $k_{d,M_2}$ represents the death rate of $\hbox {M}_2$ macrophages. We neglect source terms due to $\hbox {M}_2$ proliferation and for $\hbox {M}_2$ extravasation from vasculature.

#### Interstitial fluid

The interstitial fluid serves as a passive medium that occupies the space between cells, known as the interstitium. It becomes enriched with necrotic material due to cell death, and absorbed during cell proliferation and angiogenesis. A fundamental modeling assumption is that the only external source of mass is due to extravasation of $\hbox {M}_1$ macrophages. Unlike most multiphase models, the volume occupying phases do not sum to 1 (for $t>0$) due to macrophage extravasation. Consequently, the production term for interstitial fluid, $q_{int}$, is formulated as follows (balancing the rest of the contributions):

10 $$\begin{aligned} q_{int}= - q_h - q_c - q_v - q_{M_2} - \overline{q_{M_1}}. \end{aligned}$$

### Momentum balance equations

We assume that the flow of the cellular species in the tissue has a Reynolds number $Re<1$, and is characterized as creeping. Hence, inertial terms are neglected. The momentum balance for each cellular phase, *i*, is given by Hubbard and Byrne (2013):

11 $$\begin{aligned} \nabla \cdot \left( \theta _i \varvec{\sigma }_i \right) + \vec {F}_i = \vec {0}, \text{ for } i=h,c,v,M_1,M_2,int. \end{aligned}$$

Here, $\varvec{\sigma }_i$ denotes the stress tensor of phase, *i*, and $\vec {F}_i$ represents the forces exerted on phase *i* due to interactions with the other phases of the form. We view the volumetric phases as viscous fluids with stress tensors:

12 $$\begin{aligned} \varvec{\sigma }_i = -p_i \varvec{I} + \mu _i \left( \nabla \vec {u}_i + \left( \nabla \vec {u}_i \right) ^T \right) -\frac{2}{3} \mu _i \left( \nabla \cdot \vec {u}_i \right) \varvec{I}, \end{aligned}$$

where $\mu _i$ denotes the dynamic viscosity of phase *i*.

The momentum source term $\vec {F}_i$ is formulated as follows:

13 $$\begin{aligned} \vec {F}_i = p_i \varvec{I} \nabla \theta _i + \sum _{j,j\ne i} d_{i,j} \theta _i \theta _j \left( \vec {u}_j - \vec {u}_i \right) , \text{ for } i=h,c,v,M_1,M_2,int. \end{aligned}$$

The first term captures the pressures, $p_i$, effect on the phase’s surface, with $\varvec{I}$ being the $2\times 2$ identity matrix. Relative movement of phases *i* and *j* produces inter-phase drag as mentioned above, with drag coefficient $d_{i,j}$.

We close Eq. (11) by imposing the following boundary conditions on $\partial \Omega$:

14 $$\begin{aligned} \varvec{\sigma }_i \cdot \vec {n} = \vec {0}, \text{ for } i=h,c,v,M_1,M_2. \end{aligned}$$

To ensure a unique solution, we also prescribe the velocity of the interstitial fluid on the boundary, $\partial \Omega$, so that:

15 $$\begin{aligned} \vec {u}_{int} = \vec {0}. \end{aligned}$$

Having calculated the phase concentrations and velocities, it remains to determine the phase pressures. For this purpose, the continuity equation is formulated by summing the mass balance equation for each phase *i* $\left( i=h,c,v,M_1,M_2,int\right)$ , as formulated by Eq. (1):

16 $$\begin{aligned} \begin{aligned} \frac{\partial }{\partial t}\sum _{i}\theta _i +&\sum _{i} \nabla \cdot \left( \theta _i \vec {u}_i \right) \\&+\chi _l \nabla \cdot \left( \theta _c \nabla l \right) +\chi _g \nabla \cdot \left( \theta _v \nabla g \right) +\chi _a \nabla \cdot \left( \theta _{M_1} \nabla a \right) +\chi _b \nabla \cdot \left( \theta _{M_2} \nabla b \right) \\&= k_{ext,M_1}\theta _v a. \end{aligned} \end{aligned}$$

By computing $p_{int}$ through Eq. (16), equations of state are formulated to relate the different pressures. Here, $p_{v}$ is set equal to $p_{ref}=0$ and for the remaining pressures, we define (Hubbard and Byrne 2013):

17 $$\begin{aligned} p_h = p_c = p_{M_1} = p_{M_2} = p_{int} + \Sigma \left( \theta _h + \theta _{c} + \theta _{M_1} + \theta _{M_2} \right) . \end{aligned}$$

The function $\Sigma (\theta )$ is introduced to describe the pressure buildup due to local cell density exceeding the naturally established density in the tissue. If the natural cell density is denoted as $\theta ^*$, function $\Sigma$ is defined as follows:

18 $$\begin{aligned} \Sigma (\theta ) = {\left\{ \begin{array}{ll} \frac{\Lambda (\theta -\theta ^*)}{\sum _{i}^{6}\left( \theta _i-\theta \right) ^2}, & \text{ if } \theta \ge \theta ^*\\ 0, & \text{ otherwise }. \end{array}\right. } \end{aligned}$$

Here, $\Lambda$ is a tension constant measuring the tendency of cells to restore their natural density.

### Molecular species

Since the timescales for all processes involving molecular species (minutes) are short compared to those involving the cellular phases (days), all molecular species, *J*, are assumed to be in a quasi-steady state (Ward and King 1997; Hubbard and Byrne 2013; Lampropoulos et al. 2022; Lampropoulos and Kavousanakis 2023).

The mass balance equations for the chemical species, *J*, reflect these considerations:

19 $$\begin{aligned} D_{J} \nabla ^2 J + s_{J} = 0 ,\, \text{ for } \, J=c, g, a, b, l, f. \end{aligned}$$

In Eq. (19), we assume that diffusion is the dominant mechanism for species transport and neglect transport due to advection. We denote by $s_{J}$ the net source term for chemical species *J*. Diffusion is regulated by a diffusion coefficient $D_{J}$.

The source term for oxygen, $s_c$, is formulated as follows:

20 $$\begin{aligned} s_c = k_{rep} \theta _v\left( c_v - c \right) - \sum _{i=h,c} k_{c,i} \theta _i c- \sum _{i=h,c} k_{cm,i} \theta _i \theta _{int}\frac{c}{c_p+c}. \end{aligned}$$

The first term represents the supply of oxygen from the vasculature. We assume that it is proportional to the concentration difference between $c_v$ (the concentration of oxygen inside the vessels, which is assumed to be constant) and the oxygen concentration in the tissue, *c*. $k_{rep}$ denotes the nutrient replenishment rate constant. The second term accounts for oxygen, *c*, consumption to sustain cells, where $k_{c,i},\,\left( i=h,c\right)$ denotes the consumption rate for sustaining healthy cells ($\theta _h$) and cancer cells ($\theta _c)$. The last term describes oxygen consumption for cell proliferation. It is assumed that it is an increasing, saturating function of *c*, with $k_{cm,i},\,\left( i=h,c\right)$, denoting the maximum consumption rates for healthy and cancer cells, respectively. Since cancer cells proliferate more rapidly than healthy cells, we assume that their oxygen consumption rate is correspondingly higher (Hubbard and Byrne 2013). $c_p$ is the oxygen concentration which results to the rate being half maximal.

For all the remaining chemical species, the source terms follow the same structure, consisting of a production term and two consumption terms: one corresponding to the molecule’s natural decay and one to the binding of the molecule its cognate receptors.

21 $$\begin{aligned} s_g =&k_{p,g} \left( \theta _h +\theta _c +\theta _{M_2} \right) \frac{c}{\left( c_a + c \right) ^2}&- k_{d,g} g - k_{assoc,g}\theta _v g, \end{aligned}$$

22 $$\begin{aligned} s_a =&k_{p,a} \theta _c \frac{c}{\left( c_a + c \right) ^2}&- k_{d,a} a - k_{assoc,a} \theta _{M_1} a,\end{aligned}$$

23 $$\begin{aligned} s_b =&k_{p,b} \theta _v&- k_{d,b} b - k_{assoc,b} \theta _{M_2} b,\end{aligned}$$

24 $$\begin{aligned} s_l =&k_{p,l} \theta _{M_2}&- k_{d,l} l - k_{assoc,l} \theta _c l,\end{aligned}$$

25 $$\begin{aligned} s_f =&k_{p,f} \theta _c&- k_{d,f} f - k_{assoc,f} \theta _{M_1} f. \end{aligned}$$

Here, $k_{p,J},\,\left( J=g,a,b,l,f\right)$ denote the rates at which the chemicals are produced. $k_{assoc,J}$ ($J=g$, *a*, *b*, *l*, *f*) represent the molecule consumption through binding on relevant receptors. $k_{d,J}$ ($J=g$, *a*, *b*, *l*, *f*) denote the natural decay rates. VEGF, *g*, secretion is amplified as a response to hypoxia (Darland et al. 1999; Gee et al. 2003), reflected on the quadratic term present in VEGF’s production term. It is assumed that healthy cells, cancer cells, and macrophages secrete VEGF at a rate which attains its maximum value $\frac{k_{p,g}}{4}$ when $c=c_a < c_{init}$ and decreases to zero as $c\rightarrow 0$ and $c\rightarrow \infty$. We assume that the rate at which cancer cells produce CSF-1 has the same biphasic dependence on the oxygen concentration. For simplicity, we assume that CXCL12’s, *b*, production is attributed to the vasculature since it is secreted by stromal cells surrounding the vasculature (Arwert et al. 2018).

We impose Neumann boundary conditions for all molecular species, *J*, on the domain boundary $\partial \Omega$:

26 $$\begin{aligned} \nabla J \cdot \vec {n} = 0. \end{aligned}$$

Before presenting numerical simulations, we non-dimensionalize our system. The characteristic time and length scales are chosen to be the doubling time for a healthy cell, which is roughly one day $\left( k_{m,h}^{-1}\approx 1\, \text{ day }\right)$ (Bernard and Herzel 2006) and $L_{o} = 400 \,\mu m$ respectively. The choice of characteristic length scale is based on the typical radii reached by cancer spheroids before angiogenesis commences (Hinow et al. 2009; Folkman 1975; Norton and Popel 2016).

Based on these two units we rescaled the system’s variables as follows:

$$\begin{aligned} \begin{aligned}&t'=k_{m,h} \cdot t, \quad c'=\frac{c}{c_v}, \quad l'=\frac{l}{g_v}, \quad \vec {x_i}'=\frac{\vec {x_i}}{L_o}, \quad g'=\frac{g}{g_v}, \quad f'=\frac{f}{g_v}, \quad \\&\vec {u_i}'=\frac{\vec {u_i}}{k_{m,h}L_o}, \quad a'=\frac{a}{g_v}, \quad d'=\frac{d}{d_{max}}, \quad p_i' = \frac{p_i}{\Lambda }, \quad b'=\frac{b}{g_v}. \end{aligned} \end{aligned}$$

In addition to $k_{m,h}$ and $L_{o}$, several other units are used for the non-dimensionalization of variables: $c_v$ is the assumed-constant concentration of oxygen within the vessels; $g_v$ is a typical concentration of VEGF, determined from the initial conditions described in Section 2.5. $\Lambda$ is a tension constant measuring the tendency of cells to restore their natural density. $d_{max}$ denotes the maximum concentration of the drug in the vasculature. Further details on the non-dimensionalization can be found in SI, Chapter II.

The parameters utilized in the therapy-free model are summarized in Tables 3 and 4. Table 3 includes parameter values derived from related works and Table 4 contains those parameters that appear in the basic model and relate to the action of the $\hbox {M}_1$ and $\hbox {M}_2$ macrophages. All of the parameters are presented in dimensionless form, as described in Chapter II of the SI.

**Table 3:** Summary of model parameters, their descriptions, and estimated values (Hubbard and Byrne 2013; Lampropoulos and Kavousanakis 2023)

Parameter name	Parameter value	Description
$k_{m,c}$	2.0	Cancer cell proliferation rate constant
$k_{d,h}$	0.15	Healthy cell death rate constant
$k_{d,c}$	0.075	Cancer cell death rate constant
$c_p$	0.25	Proliferation rate saturation parameter
$c_{c_1}$	0.2	Death rate tuning parameter
$c_{c_2}$	0.1	Death rate tuning parameter
$c_{a}$	0.05	Oxygen concentration yielding maximum VEGF and CSF-1 production rates
$k_{c,h}$	0.01	Oxygen consumption rate constant by healthy cells for cell sustenance
$k_{c,c}$	0.01	Oxygen consumption rate constant by cancer cells for cell sustenance
$k_{cm,h}$	0.1	Oxygen consumption rate constant by healthy cells for mitosis
$k_{cm,c}$	$k_{cm,h}\cdot k_{m,c}$	Oxygen consumption rate constant by cancer cells for mitosis
$k_{occ}$	0.1	Vascular occlusion rate constant
$p_{crit}$	0.3	Critical pressure threshold
$\epsilon$	0.01	Interstitial fluid’s saturation parameter for angiogenesis
*h*	0.2	Vascular occlusion smoothness parameter
$\mu _i$	10.0	Dynamic viscosity for phase *i*
$d_{i,j}$	1.0	Drag between phases *i* and *j*
$\Lambda$	0.1	cellular tension constant
$d_v$	1.0	Oxygen diffusion coefficient
$D_g$	0.05	VEGF diffusion coefficient
$k_{d,g}$	0.0264	VEGF decay rate constant
$k_{assoc,g}$	$6.57\cdot 10^{-6}$	VEGF association rate constant

**Table 4:** List of parameters associated with the default model

Parameter name	Parameter value	Description	Source
$\theta _M$	0.1	Cancer cell-macrophage interaction saturation constant	-
$k_{c,M_1}$	0.2	Cancer cell - $\hbox {M}_1$ interaction rate constant	-
$k_{c,M_2}$	5.0	Cancer cell - $\hbox {M}_2$ interaction rate constant	-
$k_{d,M_1}$	0.075	$\hbox {M}_1$ death rate constant	(Parihar et al. 2010)
$k_{d,M_2}$	0.075	$\hbox {M}_2$ death rate constant	(Parihar et al. 2010)
$k_{aa}$	0.5	Alternative activation rate constant	-
$k_{ext,M_1}$	2.0	Macrophage migration rate constant	-
$D_a$	0.11	CSF-1 diffusion coefficient	(Bull and Byrne 2023)
$D_b$	0.001	CXCL12 diffusion coefficient	(Bull and Byrne 2023)
$D_l$	0.11	EGF diffusion coefficient	(Bull and Byrne 2023)
$D_f$	0.5	TGF-$\beta$ diffusion coefficient	(Bull and Byrne 2023)
$\chi _{g}$	0.14	Chemotaxis constant for chemoattraction due to VEGF	SI Section I
$\chi _{a}$	0.14	Chemotaxis constant for chemoattraction due to CSF-1	-
$\chi _{l}$	0.14	Chemotaxis constant for chemoattraction due to EGF	-
$\chi _{b}$	$5\cdot \chi _{a}$	Chemotaxis constant for chemoattraction due to CXCL12	-
$a_p$	0.25	Macrophage migration saturation parameter	-
$f_p$	0.015	Alternative activation saturation parameter	-
$k_{p,g}$	0.00959	VEGF production rate constant	SI Section I
$k_{p,a}$	0.00959	CSF-1 production rate constant	-
$k_{d,a}$	0.0216	CSF-1 decay rate constant	(Bull and Byrne 2023)
$k_{assoc,a}$	$8.75\cdot 10^{-6}$	CSF-1 association rate constant	-
$k_{p,b}$	0.54806	CXCL12 production rate constant	SI Section I
$k_{d,b}$	$0.2273\cdot 10^{-2}$	CXCL12 decay rate constant	(Bull and Byrne 2023)
$k_{assoc,b}$	$7\cdot 10^{-6}$	CXCL12 association rate constant	-
$k_{p,l}$	0.00959	EGF production rate constant	-
$k_{d,l}$	0.0216	EGF decay rate constant	(Bull and Byrne 2023)
$k_{assoc,l}$	$6.93\cdot 10^{-6}$	EGF association rate constant	-
$k_{p,f}$	0.00959	TGF-$\beta$ production rate constant	-
$k_{d,f}$	0.436	TGF-$\beta$ decay rate constant	(Bull and Byrne 2023)
$k_{assoc,f}$	$7\cdot 10^{-6}$	TGF-$\beta$ association rate constant	-

### Initial conditions

To establish the system’s initial conditions, we simulate the steady state condition of a healthy tissue. Consequently, we define the following conditions reflecting a healthy tissue’s homeostatic state:

- The volume fraction of healthy cells is set equal to $\theta _h\left( \vec {x},t=0\right) =\theta ^*=0.6$.
- The tissue devoid of cancer cells: $\theta _c\left( \vec {x},t=0\right) =0$.
- All present chemical species (oxygen and VEGF) are uniformly mixed.
- CSF-1, CXCL12, EGF, and TGF-$\beta$ are molecules associated with cancerous inflammation and thus are absent in the healthy tissue.
- There is no inflammation and no inter-cellular stresses: $\vec {u}_i=0,\,p_i=0$.
- Undifferentiated monocytes (macrophage progenitors) are assumed to be present in extremely low concentrations within the tissue, hence we practically select as initial conditions: $\theta _{M_1}\left( \vec {x},t=0\right) = \theta _{M_2}\left( \vec {x},t=0\right) = 0$.

As more thoroughly explained in SI Section I, the system can be considered closed. Consequently, the homeostatic tissue maintains a constant volume and the non-dimensionalized concentrations of the cellular phases are equivalent to their volume fractions. Based on this premise, we can simplify the calculation of $\theta _{int}$:

27 $$\begin{aligned} \begin{aligned} \sum _i^6 \theta _i\left( x,y,0 \right) = 1 \xrightarrow []{\theta _h\left( x,y,0 \right) =\theta ^*} \theta _v\left( x,y,0 \right) +\theta _{int}\left( x,y,0 \right) = 0.4. \end{aligned} \end{aligned}$$

Combining the aforementioned considerations results in the following (non-dimensionalized) system:

28 $$\begin{aligned} {\left\{ \begin{array}{ll} \theta _{int}\frac{c}{c_P+c} - k_{d,h}\frac{c_{c_1}+c}{c_{c_2}+c} = 0 \\ k_{ang}g\frac{\theta _{int}}{\epsilon +\theta _{int}} - k_{occ}\mathcal {H}\left( -p_{crit}, h \right) = 0 \\ \theta _v + \theta _{int} = 0.4 \\ \theta _v \left( 1 - c \right) -k_{c,h} \theta _h c - k_{cm,h} \theta _h \theta _{int} \frac{c}{c_p+c} = 0 \\ k_{p,g}\theta _h\frac{c}{\left( c_a + c\right) ^2} - k_{assoc,g}\theta _v g - k_{d,g} g = 0. \end{array}\right. } \end{aligned}$$

Solving the above yields the system’s equilibrium state. To compute a unique solution for the developed system, we view $k_{ang}$ as an unknown parameter. $k_{ang}$ is a parameter whose value is challenging to determine experimentally so, it is considered an unknown parameter and solved for in Eq. (28): $k_{ang}=4.87\cdot 10^{-3}$. Initiating a cancerous seed centered at $\left( x,y\right) =\left( 0, 0\right)$ with a radius $r_o=1$, results in the initial conditions for all utilized variables. The non-zero initial conditions are summarized in Table 5.

**Table 5:** Initial conditions of the model’s variables

Variable	Value/expression	Description
$\theta _h$	$0.6 - \theta _{c} (x,y,0)$	Healthy cells dimensionless concentration
$\theta _{c}$	$${\left\{ \begin{array}{ll} 0.05 \cos ^2 \left( \frac{\pi r}{2} \right) , & \text{ if } \sqrt{x^2+y^2} \le R_o = 1\\ 0, & \text{ otherwise }. \end{array}\right. }$$	Cancer cells dimensionless concentration
$\theta _{v}$	0.0175	Vessels dimensionless concentration
$\theta _{int}$	0.3825	Interstitial fluid dimensionless concentration
*c*	0.25	Oxygen’s dimensionless concentration
*g*	0.6	VEGF’s dimensionless concentration

## Methods

Numerical simulations were performed using the Comsol Multiphysics ^®^ software employing the Finite Elements Method (FEM). The computational domain is a circular disk, discretized with an unstructured mesh generated using Delaunay triangulation. The disk has a radius of 30 dimensionless units, and all computations are executed in non-dimensionalized units. Details of the non-dimensionalization process can be found in SI Section II.

All simulations are initiated by embedding one or more cancerous lesions within a healthy tissue. For a default simulation involving a tumor originating from a single seed and with both macrophage phenotypes present in its micro-environment, the model solves approximately 250, 000 degrees of freedom. It takes approximately 100 hours of computational time on an AMD Ryzen 9 3900X 12-Core Processor to simulate 300 dimensionless time units (corresponding to approximately 300 days tumor growth).

## Therapy-free model results

In this section, we present numerical results for the therapy-free model. We focus initially on the basic model which describes the growth of a small cluster of cancer cells located at the domain’s center. We then investigate the impact of varying key model parameters on the system dynamics, and finally present simulation results in which multiple tumor lesions merge. Since all computations are performed in dimensionless units, unless stated otherwise, all results are presented in dimensionless form without the need for the "$\prime$" and "*" symbols as presented in the SI.

**Fig. 3:**
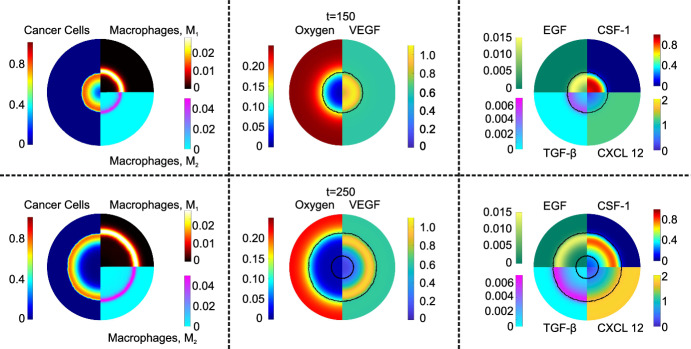
Basic model spatial distributions at dimensionless times: $t=150$ (first horizontal row) and $t=250$ (second row). The left panels depict the spatial distributions of the cancer cells and macrophages species: the left half-disk shows cancer cells, the right-upper disk depicts $M_1$ macrophages and the right-bottom disk shows $M_2$ macrophages. The middle panel shows the oxygen distribution (left half-disk) and the VEGF concentration distribution (right half-disk). The right panel shows the spatial distributions of EGF (top left quadrant), CSF-1 (top right quadrant), TGF-$\beta$ (bottom left quadrant) and CXCL 12 (bottom right quadrant). The black lines denote the tumor’s boundaries (Color figure online)

A key feature of the model is its ability to determine the spatial distributions of each cellular phase and chemical species. In the left panel of Fig. 3, we present the distributions of cancer cells and macrophages at dimensionless times $t=150$ and $t=250$, for a tumor originating from a single cancerous lesion. Although at $t=250$ there are visibly large regions where the cancer cell density is low, the total number of cancer cells continues to increase (see also the blue line in Fig. 5, which shows the evolution of the average cancer cell density). This behavior arises because, as the tumor expands approximately radially, the cancer cells proliferate in an expanding outer annulus (occupying a larger area), while the increasing tumor size exacerbates nutrient diffusion limitations, leading to progressive enlargement of the necrotic core. Of particular interest is the ability of each macrophage phenotype to infiltrate the tumor. As expected, the distribution of $\hbox {M}_1$ macrophages is broader than the distribution of $\hbox {M}_2$ macrophages. The $\hbox {M}_1$ macrophages are concentrated around the tumor’s periphery. When they infiltrate the tumor, they are exposed to TGF-$\beta$ which triggers alternative activation. Consequently, the $\hbox {M}_2$ macrophages are concentrated near the tumor’s outer boundary.

Complementing the cancer and macrophage distributions, the middle and right panels of Fig. 3 depict the distributions of the chemical species, at $t=150$ and $t=250$. To provide context on their relative positions within the domain, we indicate the tumor’s boundary (and the necrotic boundary) as a black line. These boundaries are defined as the contour where $\theta _c=0.01$.

The plots of oxygen distribution (left half-disk in the middle panels of Fig. 3) show how the tumor’s expansion leads to vascular occlusion, which reduces tissue oxygenation. As the tumor grows, oxygen diffusion becomes insufficient to meet the tissue’s demands, and hypoxic areas form (light and medium dark blue). Hypoxia drives the production of VEGF and CSF-1, their concentrations peaking in the hypoxic regions. In contrast, concentrations of EGF and TGF-$\beta$ are greatest within the tumor’s well-oxygenated, proliferative zone. The elevated cancer cell concentrations in this region enhance production of CXCL12. Due to its slow rate of diffusion, CXCL12 remains localized near its site of production.

Motivated by the numerical results for the basic model, we now investigate how macrophages can inhibit or facilitate tumor growth. We consider three scenarios: (i) a tumor where both macrophage phenotypes are present; (ii) a tumor where only the $\hbox {M}_1$ phenotype is expressed, and (iii) a tumor where both $\hbox {M}_1$ and $\hbox {M}_2$ macrophages are present (they occupy space) but they are inert.

**Fig. 4:**
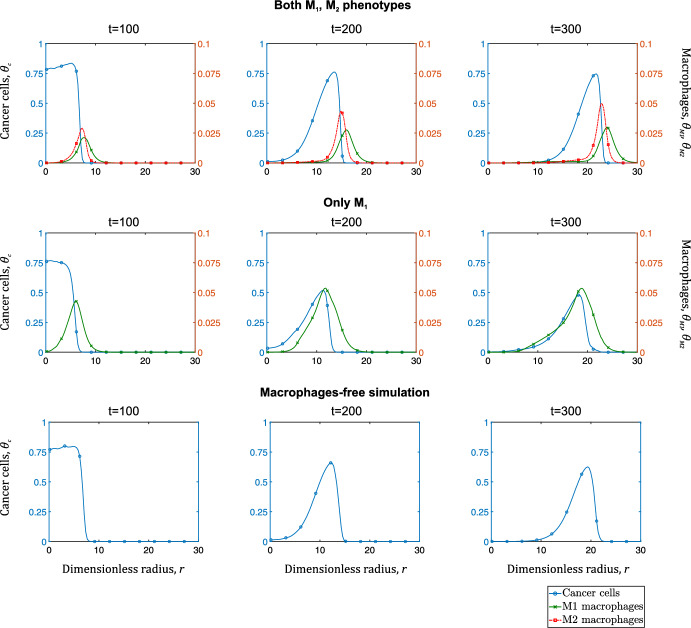
Series of plots showing the average radial distributions of cancer cells (left y-axis - blue curve with circles), $\hbox {M}_1$ macrophages (right y-axis - green crossed curve), and $\hbox {M}_2$ macrophages (right y-axis - red dotted curve with squares) at times $t=100, 200, 300$ for three scenarios. The top row corresponds to a tumor in which both macrophage phenotypes are present and functional; the middle row depicts a scenario where only $\hbox {M}_1$ macrophages are present (and alternative activation cannot occur), and the bottom row corresponds to a tumor whose macrophages are inert (Color figure online)

We compare the spatial distributions of cancer and immune cells for each case. In Fig. 4, we present the average radial distributions of cancer cells and macrophages at dimensionless times $t=100$, 200, and 300. We observe that when both $\hbox {M}_1$ and $\hbox {M}_2$ macrophages are active, the cancer cells invade most rapidly. Secondly, the radial distribution of the $\hbox {M}_1$ macrophages is broader as they enter the system via the vascular network and migrate towards the tumor. Conversely, $\hbox {M}_2$ macrophages penetrate deeper into the tumor and localize in hypoxic regions, where they promote tumor cell proliferation.

To quantify the dynamics of the cancer cells and macrophages, we calculate the average concentration, $\overline{\theta }_i$, for $i=c,M_1,M_2$. $\overline{\theta }_i$ is calculated over the domain’s surface, *S*:

29 $$\begin{aligned} \overline{\theta }_i=\frac{1}{S}\iint _{\Omega } \theta _{i}\,dx\,dy, \; \text{ for } \; i=c,M_1,M_2. \end{aligned}$$

**Fig. 5:**
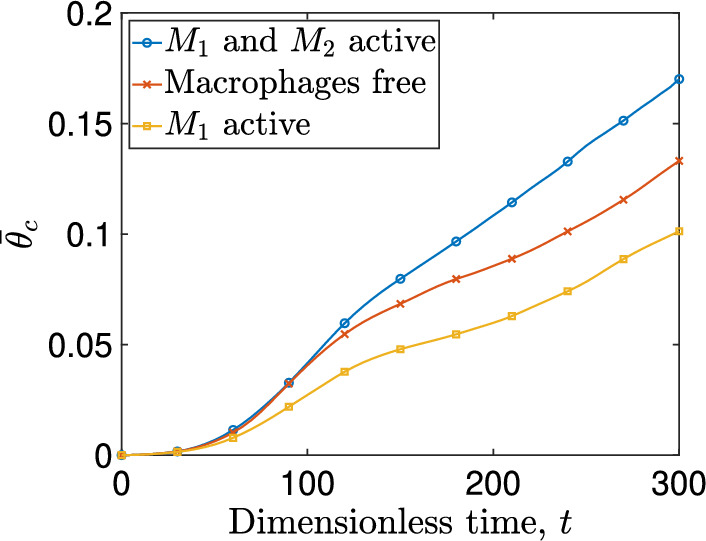
Average concentration, $\overline{\theta }_c$, of cancer cells plotted against dimensionless time for three scenarios: a tumor with both macrophage phenotypes being expressed (blue curve with circles), macrophages being inert (red crossed curve), and macrophages immune to alternative activation (yellow curve with squares) (Color figure online)

Figure 5 illustrates the average cancer cells concentration, $\overline{\theta }_c$, for the three aforementioned scenarios. If we view the scenario where macrophages are passive (case (iii)) as a reference point, we observe that the $\hbox {M}_1$ phenotype significantly inhibits tumor expansion. However, $\hbox {M}_1$ macrophages alone are unable to eradicate the tumor. By contrast, alternative activation and expression of the pro-tumor macrophage phenotype accelerate tumor growth.

**Fig. 6:**
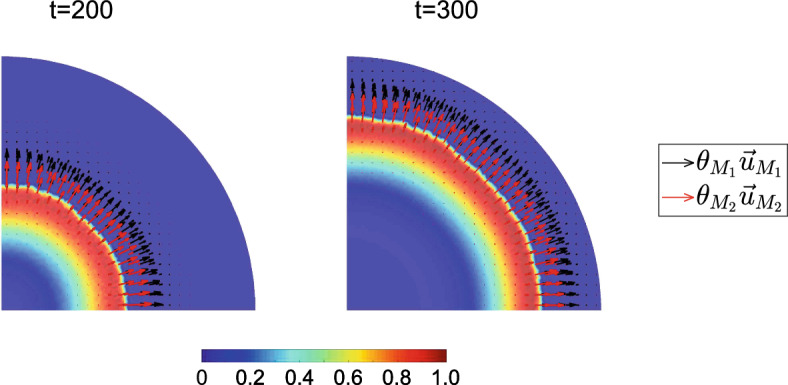
Phase flux vector distribution of macrophage phenotypes, alongside the surface distribution of cancer cells at $t=200$ (left panel) and $t=300$ (right panel). The $M_1$ phase flux vectors are represented by red arrows, and the $M_2$ phase flux vectors are depicted by black arrows (Color figure online)

In Fig. 6 we plot the phase fluxes of the two macrophage phenotypes: $\theta _{M_1}\vec {u}_{M_1}$, and $\theta _{M_2}\vec {u}_{M_2}$, together with the spatial distribution of cancer cells at $t=200$ and $t=300$. The advancing tumor front and the forces it generates drive outward movement of both macrophage phenotypes. The fluxes for $\hbox {M}_2$ macrophages (red arrows), are higher because chemotaxis drives them to regions with higher concentrations of CXCL12. The magnitude of the outward flux of $\hbox {M}_1$ macrophages is smaller because they are subject to an inward chemotactic force, which drives them to hypoxic areas which are rich in CSF-1.

### 4.0.1 Parametric analysis

We investigate the impact of varying some of the model parameters for which we lack accurate estimates. We focus on: (i) $k_{c,M_1}$, $k_{c,M_2}$ which represent the rates of interactions between cancer cells and $M_1$, $M_2$ macrophages, respectively (see Eq. (4)); (ii) $k_{aa}$ which regulates the rate of alternative activation (see Eq. (8) and Eq. (9)); (iii) $k_{ext,M_1}$ which denotes the $M_1$ macrophage migration rate constant (see Eq. (8)); (iv) $\chi _a, \chi _b$ representing the chemosensitivity of $\hbox {M}_1$ and $\hbox {M}_2$ to spatial gradients of CSF-1 and CXCL12, respectively (see Eq. (1)). We quantify the impact of varying these parameters by either comparing their spatial distributions with the basic model or, recording the temporal evolution of the cancer cell average concentration, $\overline{\theta }_c$.

**Fig. 7:**
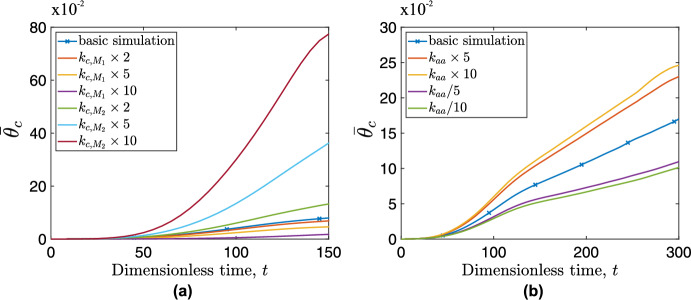
Temporal evolution of cancer cell average concentration, $\overline{\theta }_c$ for simulations differing from the basic simulation by the value of one certain parameter: (a) $k_{c,M_1}$ or $k_{c,M_2}$ and (b) $k_{aa}$. The parameter values for the basic simulation are: $k_{c,M_1}=0.2$, $k_{c,M_2}=5.0$, $k_{aa}=0.5$

Figure 7 (a) shows that increasing $k_{c,M_1}$ slows tumor growth, but does not lead to tumor eradication. In contrast, increasing $k_{c,M_2}$ increases the tumor’s growth rate. Figure 7 (b) shows that changing $k_{aa}$ by a factor of 5 significantly alters the tumor’s growth dynamics, with an increase in $k_{aa}$ promoting tumor growth and a reduction inhibiting growth. However, further increasing or reducing this factor (to $\times 10$ or $\div 10$) causes the corresponding curves to diverge less than one might anticipate. This suggests that there are limits to the attainable cancer cell average concentration, $\overline{\theta }_c$, regardless of $k_{aa}$ value; implying the existence of other limiting factors such as the rate of TGF-$\beta$ replenishment in the tissue.

**Fig. 8:**
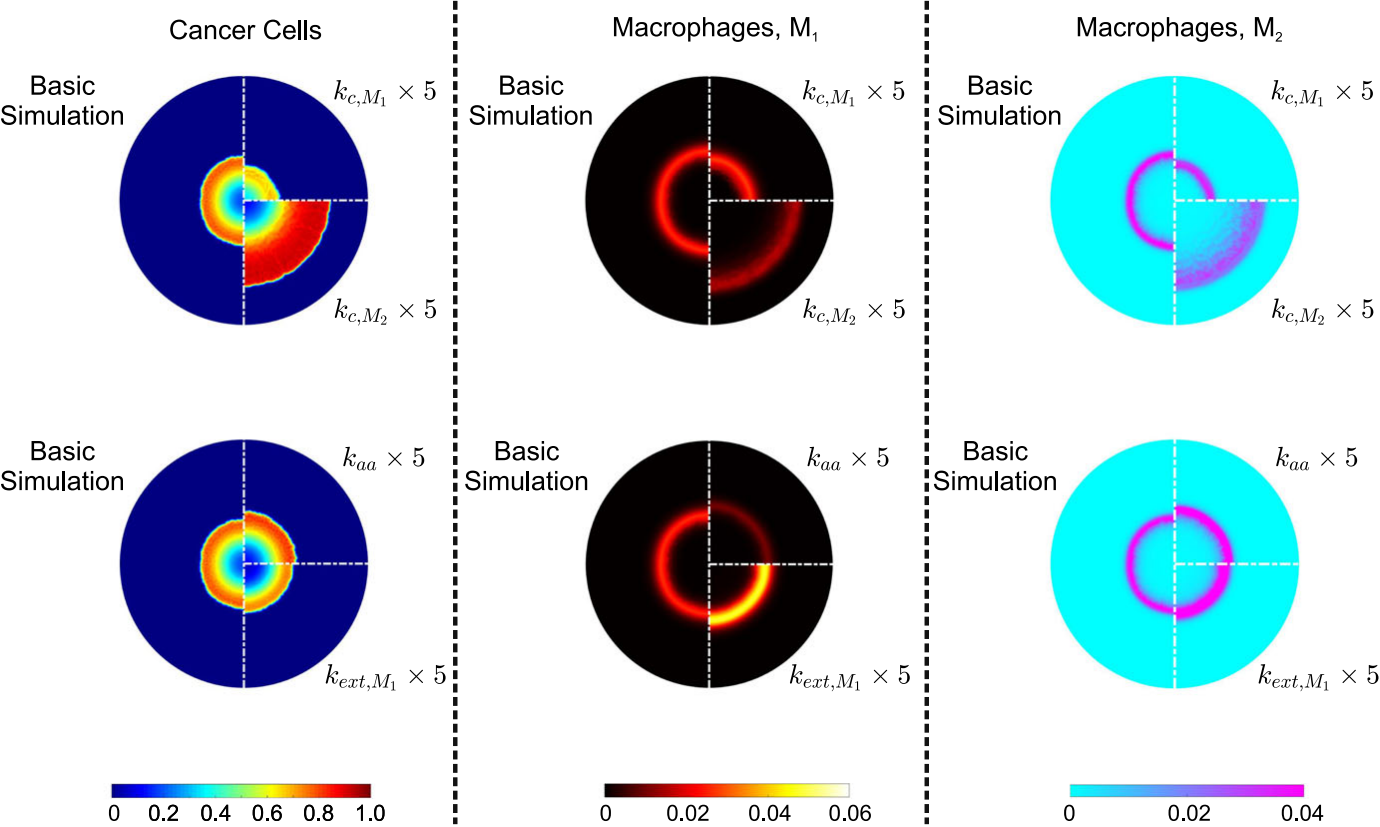
Surface distributions of cancer cells, $\theta _c$ (left column), macrophages $M_1$,$\theta _{M_1}$ (middle column), and macrophages $M_2$, $\theta _{M_2}$ (right column) at dimensionless time, $t=150$. The first row presents the impact of increasing the cancer cell-$M_1$ interaction constant, $k_{c,M_1}$, (top-right quadrant) and the cancer cell-$M_2$ interaction constant, $k_{c,M_2}$ (bottom-right quadrant) by a factor of 5 compared to the basic simulation (left half-disk). The second row shows the impact of increasing the alternative activation rate constant, $k_{aa}$ by a factor of 5 (top-right quadrant), and the effect of the migration rate constant, $k_{ext,M_1}$ when increased by a factor of 5 (bottom-right quadrant)

We now take a closer look at some simulation results by examining the spatial distributions of cancer cells and macrophages at specific time points. Figure 8 compares the distributions of cancer cells and macrophages at $t=150$ for the default case, with the distributions when $k_{aa},\,k_{c,M_1},\,k_{c,M_2}$, and $k_{ext,M_1}$ are increased by a factor of 5.

Increasing the cancer cell-$\hbox {M}_1$ interaction rate constant, $k_{c,M_1}$, significantly reduces the cancer cell density and enables the immune cells to penetrate the tumor more effectively. The subsequent reduction in cancer cells leads to lower levels of cytokines, such as TGF-$\beta$ and, hence, lower concentrations of $M_2$ macrophages. However, the increased penetration of $M_1$ macrophages results in a broader distribution of both macrophage phenotypes.

To highlight the differences between the system with enhanced cancer cell-$M_2$ reaction rate constant, $k_{c,M_2}$, and the basic case, we use snapshots taken at $t=150$ (see Fig. 8). The morphological differences between the two tumors are evident. The increased activity of pro-tumor macrophages leads to a tumor with a significantly wider and denser proliferating rim. Although the total number of immune cells in the tissue remains the same, they are distributed over a broader area due to the forces generated by the aggressive tumor growth.

Increasing $k_{aa}$ produces a more aggressive tumor, with the proliferative rim showing higher cancer cell concentrations and the phenotypic balance shifting towards pro-tumor macrophages. Lastly, we enhance the migration rate constant, $k_{ext,M_1}$; given that we are at $t=150$, which is past the initial rapid accumulation of $M_2$ macrophages, it is evident that the anti-tumor macrophages have begun to overwhelm the tumor, leading to tumor growth slowing down. Despite this, with the default parameter values (except for the increased $k_{ext,M_1}$) the anti-tumor macrophages remain largely confined to the tumor’s periphery. The influx of macrophages also naturally leads to an increase in pro-tumor macrophages (see Fig. 8).

### Multi-seed

**Fig. 9:**
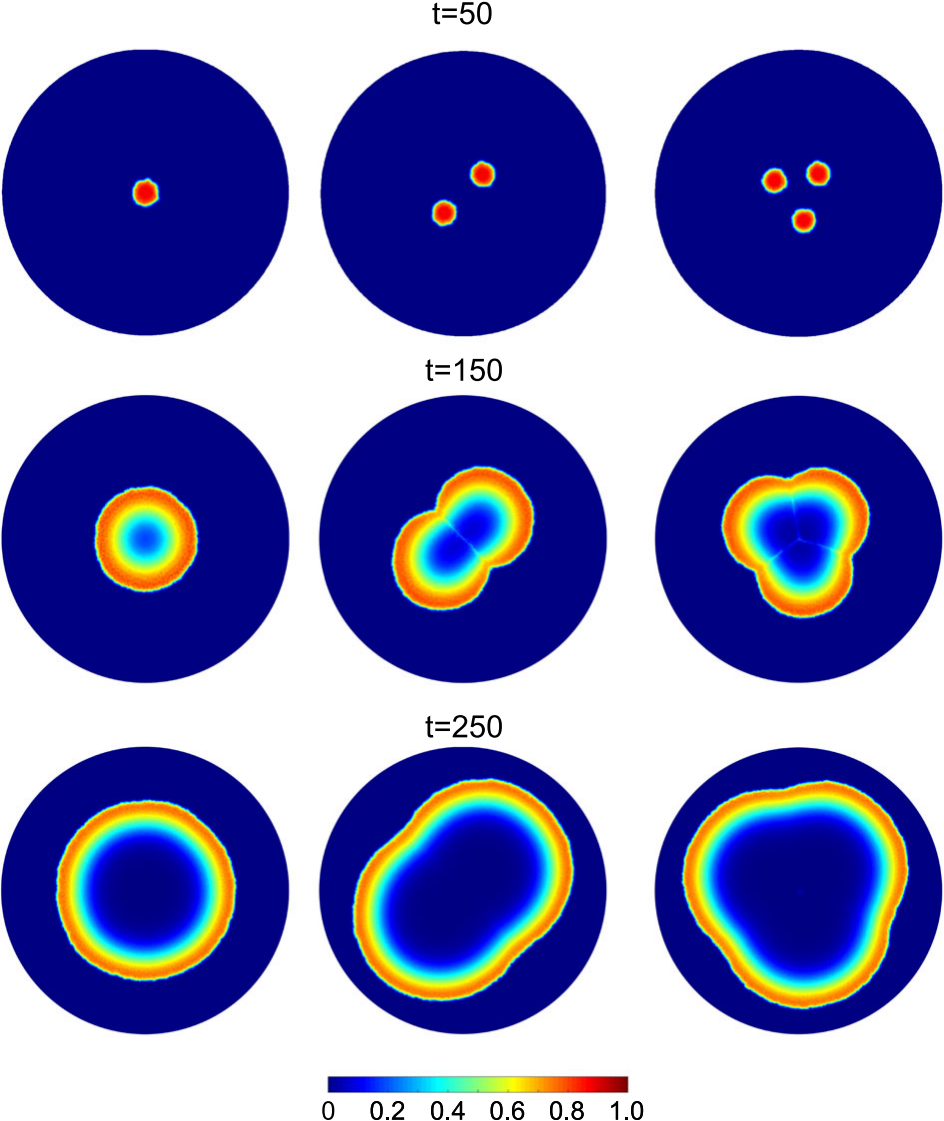
Spatial distributions of cancer cells for a tumor originating from a single (1st column), double (2nd column), and triple initial seed (3rd column) are depicted. At $t=0$, the same number of cancer cells are divided equally between one, two or three seeds. Each row corresponds to a specific time point ($t=50$, 150, and 250)

In this section, we simulate tumors originating from different numbers of initial seeds, with both macrophage phenotypes present in their micro-environment. This setup reflects evidence that tumors often originate from multiple spatially distinct lesions (Kang et al. 1997), which, upon merging, create hypoxic junctions that promote $\hbox {M}_2$ macrophage polarization (Vito et al. 2020). By varying the initial spatial distribution while keeping total tumor burden constant, we isolate how geometry-induced microenvironmental heterogeneity affects immune infiltration and tumor growth dynamics. Specifically, in Fig. 9, we show how the spatial surface distributions of cancer cells change over time for tissues seeded with one, two or three small tumors (columns 1, 2 and 3, respectively). Each simulation is initialized with the same number of tumor cells. Tumors originating from multiple seeds expand more rapidly than tumors initiated from a single lesion. Furthermore, hypoxic areas appear in the areas where the initial seeds merge.

**Fig. 10:**
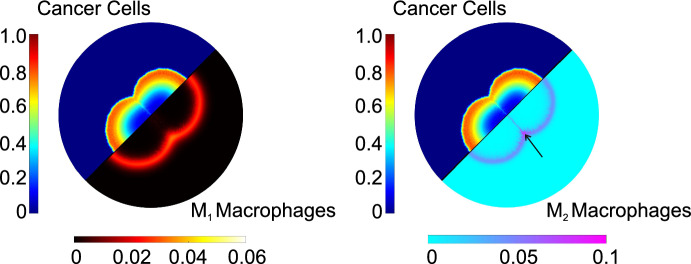
Juxtaposed spatial distributions of cancer cells with $\hbox {M}_1$ macrophages (left panel) and cancer cells with $\hbox {M}_2$ macrophages (right), for a tumor originating from a double seed at dimensionless time, $t=150$. We observe a higher concentration of $\hbox {M}_2$ macrophages at the intersection of the two tumor seeds. The arrow in the right panel indicates the region with the highest $\hbox {M}_2$ concentration (Color figure online)

These zones become significant when we analyze the corresponding distributions of macrophages; Figure 10 shows that the pro-tumor macrophages are concentrated on the tumor’s periphery and the intersections formed between the two initial spheroids. In contrast to that, $\hbox {M}_1$ macrophages are not present in this region.

**Fig. 11:**
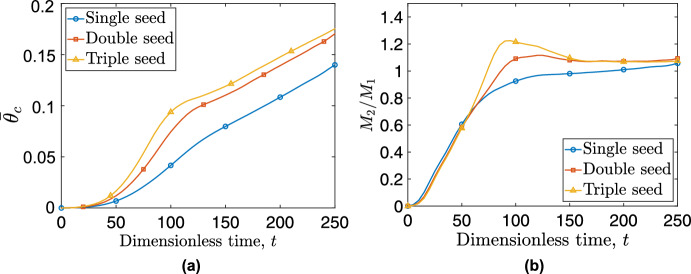
(a) Temporal evolution of cancer cell average concentration, $\overline{\theta }_c$, for tumors originating from one (blue curve with circles), two (red curve with rectangles), or three initial seeds (yellow curve with triangles). (b) Temporal evolution of the ratio of pro-tumor macrophages, $\hbox {M}_2$, to pro-inflammatory macrophages, $\hbox {M}_1$, for tumors originating from one, two or three initial seeds (Color figure online)

$\hbox {M}_2$ macrophages play a significant role in the tumor’s expansion, as evidenced in Fig. 11 (a), where we compare the cancer cell $\overline{\theta }_c$ for tumors originating from single, double and triple seeds. While all three tumors initially contain the same number of cancer cells, distributing these cells into multiple seeds significantly enhances the tumor’s growth rate. This enhancement can be attributed partly to the higher perimeter-to-surface ratio resulting from distributing the same number of cells into more seeds, especially during the earlier stages of tumor development. An additional factor is the formation of hypoxic zones at the intersections of the seeds. These zones promote the activation of the $\hbox {M}_2$ macrophage phenotype, thus contributing to tumor growth. The correlation between macrophage infiltration in hypoxic niches and a poorer prognosis is supported by experimental evidence (Vito et al. 2020).

The formation of hypoxic pockets in tumors originating from multiple initial seeds also impacts the ratio of $\hbox {M}_2$ to $\hbox {M}_1$ macrophages, a metric generally associated with a poor prognosis (Jayasingam et al. 2020). Specifically, in Fig. 11 (b) we compare the time evolution of the $\hbox {M}_2$ to $\hbox {M}_1$ ratio for tumors originating from one, two or three seeds. The localized concentration of TAMs in hypoxic niches (observed in multi-seeds simulations) causes a spike in the $\hbox {M}_2$/$\hbox {M}_1$ ratio. This ratio can increase by up to $25\%$ for a tumor originating from two seeds and up to $50\%$ for a tumor originating from three seeds, when compared to a tumor originating from one seed. We also note that as the different tumor seeds merge to form a single mass, the $M_2/M_1$ ratio asymptotes to the same value regardless of the initial number of tumor seed.

## Immunotherapy model

**Fig. 12:**
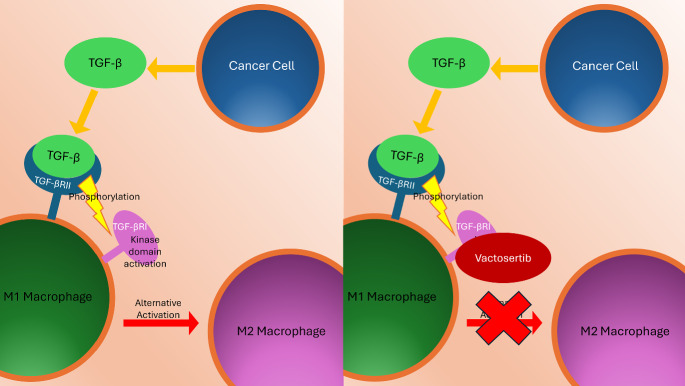
Schematic diagrams illustrating how vactosertib inhibits alternative activation of $\hbox {M}_1$ macrophages. TGF-$\beta$ produced by cancer cells binds to TGF-$\beta$ type II receptors (TGF-$\beta$RII) on $\hbox {M}_1$ macrophages, simulating the recruitment and phosphorylation of TGF-$\beta$ type I receptors (TGF-$\beta$RI). This in turn activates the kinase domain of TGF-$\beta$RI (Tzavlaki and Moustakas 2020). In the presence of vactosertib, kinase activation on TGF-$\beta$RI is inhibited, preventing its phosphorylation and the subsequent alternative activation of the $\hbox {M}_1$ macrophages (Kim et al. 2021)

We now extend our model to investigate the efficacy of a macrophage targeting immunotherapy. We focus on the drug vactosertib, which binds to TGF-$\beta$ receptors on $\hbox {M}_1$ macrophages and, in doing so, inhibits their switching to a pro-tumor phenotype (Kim et al. 2021; Pubchem 2024). In what follows, we will assume that $\hbox {M}_1$ macrophages with bound vactosertib cannot change their phenotype. A graphical representation of the drug’s mechanism of action is presented in Fig. 12.

In order to account for immunotherapy involving vactosertib, we include two new variables to our model. We denote by *d* the concentration of vactosertib and by $M_{1_p}$ those $\hbox {M}_1$ macrophages whose receptors are bound by the drug and which are, therefore, frozen in the $\hbox {M}_1$ phenotype and resistant to alternative activation by TGF-$\beta$. We describe below the required model modifications.

### Macrophages permanently exhibiting an anti-tumor phenotype

The mass balance equation for $M_{1_p}$ is similar to Eq. (1). In particular, the new sub-population exhibits the same phenotype and chemosensitivity as $\hbox {M}_1$ macrophages. Consequently, the mass balance equation for $M_{1_p}$ is:

30 $$\begin{aligned}&\frac{\partial \theta _{M_{1_p}}}{\partial t} + \nabla \cdot \left( \vec {u}_{M_{1_p}} \theta _{M_{1_p}} \right) + \chi _a \nabla \cdot \left( \theta _{M_{1_p}} \nabla a \right) \nonumber \\&\quad = k_{assoc,M_1} \theta _{M_1} \frac{d}{d_p+d} - k_{d,M_{1_p}} \theta _{M_{1_p}}. \end{aligned}$$

The source and sink terms on the right hand side of Eq. (30) represent the production of $\hbox {M}_{1_p}$ macrophages due to vactosertib binding to $\hbox {M}_1$ macrophages and removal due to natural death. The parameter $k_{assoc,M_1}$ is the maximum rate of drug binding to the TGF-$\beta$ receptors on the surface of $\hbox {M}_1$ macrophages and $d_p$ the drug concentration at which the association rate is half maximal. We assume that the vactosertib-$\hbox {M}_1$ binding is irreversible. The sink term for $\hbox {M}_{1_p}$ macrophages is identical to that for $\hbox {M}_1$ macrophages (see Eq. (8)). $k_{d,M_{1_p}}$ denotes the macrophages death rate constant (with $k_{d,M_{1_p}}=k_{d,M_1}$.

The velocity field for $\hbox {M}_{1_p}$, $\vec {u}_{M_{1_p}}$, is calculated using Eq. (11), as described in Sec. 2.3. Including a new cellular phase affects the functional form of several other model equations. The $\hbox {M}_{1_p}$ source term is balanced by a sink term for $\hbox {M}_1$ macrophages:

31 $$\begin{aligned} q_{M_1}^{new} = k_{ext,M_1} \theta _v \frac{a}{a_p+a} -k_{d,M_1} \theta _{M_1} - k_{aa} \theta _{M_1} \frac{f}{f_p+f} - k_{assoc,M_1} \theta _{M_1} \frac{d}{d_p+d}. \end{aligned}$$

The following adjustments are made in the expressions for the source terms of cancer cells, $q_c$, and vessels, $q_v$:

32 $$\begin{aligned} q_c^{new}= &  \left( k_{m,c} + k_{c,M_2} \frac{\theta _{M_2}}{\theta _M+\theta _{M_2}} \right) \theta _{c} \theta _{int} \frac{c}{c_p+c} - k_{d,c} \theta _{c} \frac{c_{c_1} + c}{c_{c_2} + c} \nonumber \\ &  - k_{c,M_1} \theta _c \frac{\theta _{M_1}+\theta _{M_{1_p}}}{\theta _M+\theta _{M_1}+\theta _{M_{1_p}}}, \end{aligned}$$

33 $$\begin{aligned} q_v^{new}= &  k_{ang} \theta _{v} g \frac{\theta _{int}}{\epsilon + \theta _{int}} - k_{occ} \theta _{v} \mathcal {H} \left( p_{cell}^{new} - p_{crit}, h \right) , \end{aligned}$$

where

34 $$\begin{aligned} p_{cell}^{new}=\frac{1}{\sum _{i}^{6}\theta _i} \left( \theta _h p_h+\theta _c p_c+\theta _{M_1} p_{M_1}+\theta _{M_{1_p}} \cdot p_{M_{1_p}}+\theta _{M_2} p_{M_2}\right) , \end{aligned}$$

and $$p_{M_{1_p}}=p_{M_1}$$.

### Immunotherapeutic drug

In clinical trials vactosertib is administered orally twice a day (Samsung Medical Center (Responsible Party 2024)) and reaches the tumor via the vasculature. Vactosertib’s mass balance follows the formulation presented in Eq. (19). The net production term $s_d$ accounts for drug delivery, its removal due to binding to anti-tumor macrophage receptors, and removal due to natural decay:

35 $$\begin{aligned} s_d = k_{rep,d} \theta _v \left( d_v -d\right) -k_{assoc,d} \left( \theta _{M_1}+\theta _{M_{1_p}}\right) \cdot d - k_{d,d} \cdot d. \end{aligned}$$

Here, $k_{rep,d}$ denotes the drug delivery rate via the vasculature. $k_{assoc,d}$ is the rate at which the drug binds irreversibly to TGF-$\beta$ receptors on anti-tumor macrophages and, $k_{d,d}$ denotes its rate of natural decay. We denote by $d_v=d_v\left( t\right)$ the time-dependent drug concentration in the vasculature. The drug concentration in the vasculature takes its maximum value, $d_{max}$, upon each oral administration and decays exponentially between doses:

36 $$\begin{aligned} d_v\left( t\right) = d_{max} \sum _{i=1}^{N} H\left( t-t_{pill,i}\right) \cdot e^{-k_{el,d}\left( t-t_{pill,i}\right) }. \end{aligned}$$

Here, $t_{pill,i}$ is the time at which the *i*-th dose is delivered and

37 $$\begin{aligned} t_{pill,i}= (i-1)T_{pill}+t_{0}^{pill}, \; i=1,...,N. \end{aligned}$$

Where $t_{0}^{pill}$ is the time at which treatment starts. $T_{pill}$ is the time between doses and *N* is the number of doses administered. Additionally, $k_{el,d}$ denotes the drug elimination rate within the vasculature and *H* denotes the Heaviside step function.

### Parameters and parameter derivation

The parameters utilized in the immunotherapy model are presented in Table 6. All parameters are dimensionless (see Section II of the SI for details).

**Table 6:** List of parameters associated with immunotherapy

Parameter name	Parameter value	Description	Source
$k_{d,M_{1_p}}$	0.075	$M_{1_p}$ death rate constant	(Parihar et al. 2010)
$D_d$	0.231	Drug diffusion coefficient	SI Section I
$d_p$	$3\cdot 10^{-3}$	Immunotherapy’s saturation parameter	-
$k_{rep,d}$	0.25	Drug’s replenishment rate constant	-
$k_{d,d}$	0.016	Drug’s decay rate constant	-
$k_{assoc,d}$	0.016	Drug’s association rate constant	(Jung et al. 2020)
$k_{assoc,M_1}$	2.0	$\hbox {M}_1$ - drug association rate constant	-
$k_{el,d}$	4.8	Drug’s elimination from plasma rate constant	(Jung et al. 2020)
$T_{pill}$	0.5	Drug’s administration period	(Jung et al. 2020; Samsung Medical Center (Responsible Party 2024))
$t^{pill}_0$	100	Drug’s initial administration time	-
*N*	10	Number of pills administered	(Samsung Medical Center (Responsible Party 2024))

## Immunotherapy model results

In this section, we present results from numerical simulations of our immunotherapy model. Vactosertib has shown promise in clinical trials (Malek et al. 2023; Lee 2020; Metropulos et al. 2022), and additional clinical trials are currently underway (Samsung Medical Center (Responsible Party (2024); Case Comprehensive Cancer Center (Responsible Party) (2024a, 2024b); Yonsei University (Responsible Party) (2024)).

**Fig. 13:**
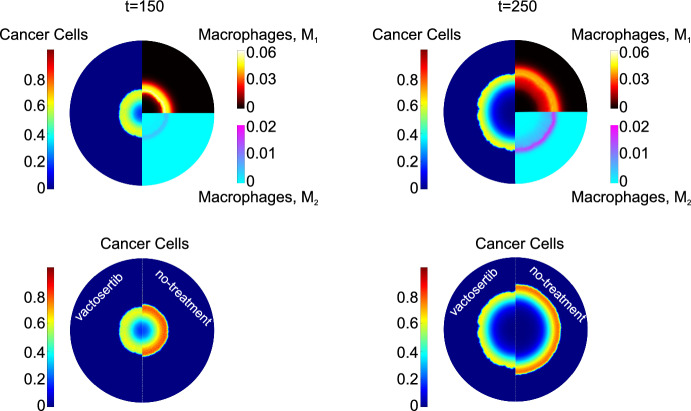
First horizontal line: Surface distributions of cancer cells (left half-disk), $M_1$ macrophages (upper-right quadrant), and $M_2$ macrophages (bottom-right quadrant), at dimensionless times $t=150$ (left column), and $t=250$ (right column) for a tumor treated with immunotherapy. Immunotherapy is initiated at dimensionless time, $t_0^{im}=100$. Second row: Surface distributions of cancer cells for a tumor treated with vactorsertib (left half-disk) and an untreated tumor (right half-disk). The left column shows results at dimensionless time $t=150$, and the right column shows the comparison at dimensionless time $t=250$

For this computational study, we initiate immunotherapy at $t^{pill}_0=t_0^{im}=100$ (see Eq. (36) and (37)). For comparison with the base case (Fig. 3), in Fig. 13 we plot the spatial distribution of cancer cells and macrophages at dimensionless times $t=150$ and $t=250$. We observe a significant delay in tumor growth (see distributions on left half of Fig. 13). The density of anti-tumor macrophages is larger when immunotherapy is applied, and the density of $\hbox {M}_2$ macrophages is reduced. The anti-tumor macrophages increase in number and adopt a broader distribution, as the drug prevents them from switching to an $M_2$ phenotype.

**Fig. 14:**
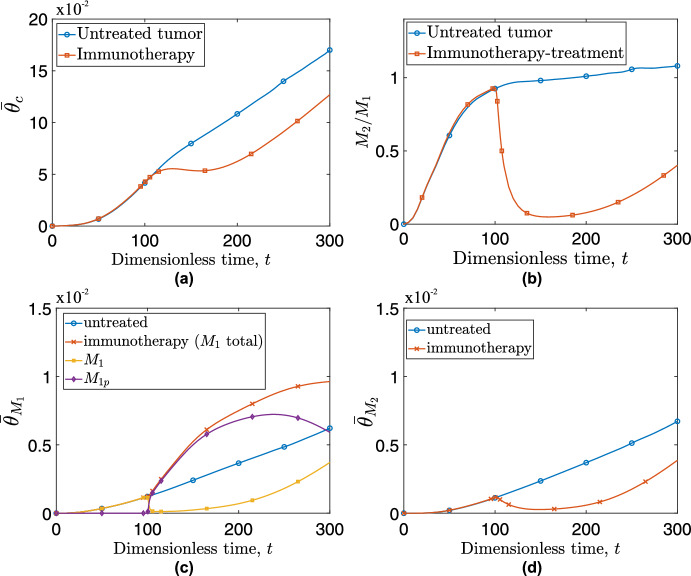
Evolution of (a) cancer cells average concentration, $\overline{\theta }_c$, and of (b) the ratio of pro-tumor macrophages, $\hbox {M}_2$ to pro-inflammatory macrophages, $\hbox {M}_1$ for an untreated tumor (blue curve with circles) and a tumor treated with immunotherapy (red curve with squares). Evolution of average concentration of (c) $\hbox {M}_1$ and (d) $\hbox {M}_2$ macrophages for an untreated tumor (blue lines with circles) and an immunotherapy-treated tumor (red crossed lines). For the immunotherapy-treatment simulation, panel (c) also illustrates the evolution of drug-bound ($M_{1p}$ - purple line with diamonds) and unbound ($\hbox {M}_1$ - yellow line with squares) macrophages (Color figure online)

Figure 14 (a) shows the impact of immunotherapy on the average concentration, $\overline{\theta }_c$, of cancer cells. Immunotherapy successfully halts cancer cell proliferation for a time interval of approximately $\Delta t \approx 80$ dimensionless time units (equivalent to a few months). At longer times, however, the tumor reverts to its initial growth rate due to the drug concentration’s decline in the tissue.

Figure 14 (b) shows that immunotherapy significantly reduces the $M_2/M_1$ ratio by up to $95\%$ for an extended time interval. However, as the drug’s effect begins to diminish (after a few months), the $\hbox {M}_2$/$\hbox {M}_1$ macrophage ratio increases and the tumor relapses. When calculating $\hbox {M}_2$/$\hbox {M}_1$ ratios, we use the sum of $\hbox {M}_1+$ $\hbox {M}_{1_p}$ as a denominator. It is also of interest to examine the number of macrophages in the tissue. To achieve this, in Fig. 14 (c) and (d) we compare the dynamics of the $\overline{\theta }_i$ metrics for $\hbox {M}_1$ and $\hbox {M}_2$ macrophages for an untreated tumor (the basic model) with the dynamics for a tumor treated with vactosertib. Upon administration of the drug (at $t=100$), we observe a rapid decline in the $\hbox {M}_2$ phenotype, because the drug inhibits alternative activation of $\hbox {M}_1$ macrophages. At the same time, being immune to alternative activation, $\hbox {M}_1$ macrophages continue to rise in numbers, contributing to the sudden drop in the $M_2/M_1$ ratio observed in Fig. 14 (b). However, the circulatory system continues to serve as a pathway for newly recruited macrophages to enter the system. These macrophages continue to consume the administered drug and provide more receptors for TGF-$\beta$. Indeed, at dimensionless time, $t\approx 180$, the population of ${M_2}$ macrophages relapses and continues to increase thereafter. Gradual depletion of the drug slows the growth rate of drug-bound macrophages, and at dimensionless time $t\approx 235$ we observe a decline in $M_{1p}$. As the drug levels decline, the $\overline{\theta }_i$ for both $\hbox {M}_1$ and $\hbox {M}_2$ macrophages adopt profiles similar to those observed in the untreated tumor.

**Fig. 15:**
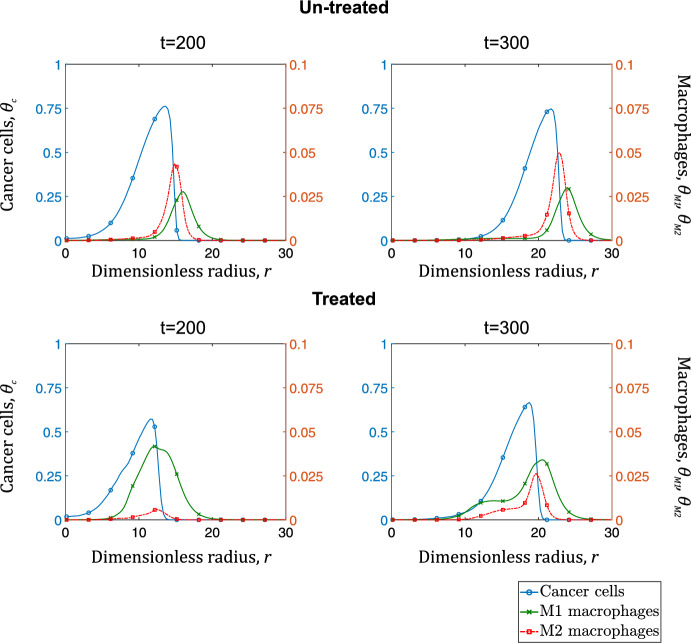
Average radial distributions of cancer cell concentrations (left y-axis - blue curve with open circles), $\hbox {M}_1$ macrophage concentrations (right y-axis - green crossed curve), and $\hbox {M}_2$ macrophage concentrations (right y-axis - red dotted curve with squares). Each column represents a specific time point ($t=200$, and 300). Top row shows the radial distributions for an untreated tumor, and the bottom row depicts distributions for a tumor treated with immunotherapy (Color figure online)

In Fig. 15, we compare the average radial distributions of cancer cells and macrophages for the untreated and immunotherapy-treated tumors (rows 1 and 2 respectively) at dimensionless times, $t=200$, and 300. For the treated tumor, we consider $\hbox {M}_1$ macrophages as the sum of $\hbox {M}_1+$ $\hbox {M}_{1_p}$ as both have anti-tumor properties. We observe that $\hbox {M}_1$ macrophages, not only increase significantly in numbers in the presence of the immunotherapy drug but also gradually infiltrate the spheroid, resulting in a more uniform anti-tumor effect. Having said that, at $t=300$, when the effect of the drug has largely dissipated, the profile of cancer cells looks similar for the treated and untreated cases.

### Parametric analysis

**Fig. 16:**
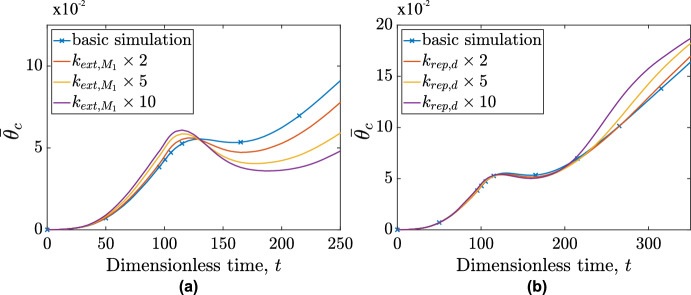
Temporal evolution of cancer cell average concentration, $\overline{\theta }_c$, for scenarios differing from the basic immunotherapy (basic simulation) case by varying one parameter: (a) the macrophage migration rate constant, $k_{ext,M_1}$ and (b) the drug replenishment rate, $k_{rep,d}$

In this section, we conduct a second parametric study, focusing initially on the parameters $k_{ext,M_1}$ (macrophage migration rate constant), and $k_{rep,d}$ (drug’s replenishment rate constant). Figure 16 shows how the dynamics of the cancer cell average concentration, $\overline{\theta }_c$, change as both parameters vary. In Fig. 16 (a), we observe that increasing $k_{ext,M_1}$ increases the cancer’s growth rate because the increased influx of $\hbox {M}_1$ macrophages leads to a higher proportion of $\hbox {M}_2$ macrophages. However, the effect is not significant. After the introduction of immunotherapy $\left( t\ge 100\right)$, higher $k_{ext,M_1}$ values cause a shift toward an anti-tumor $M_1$ phenotype, driving a reduction in the tumor growth rate which becomes more pronounced as $k_{ext,M_1}$ increases.

**Fig. 17:**
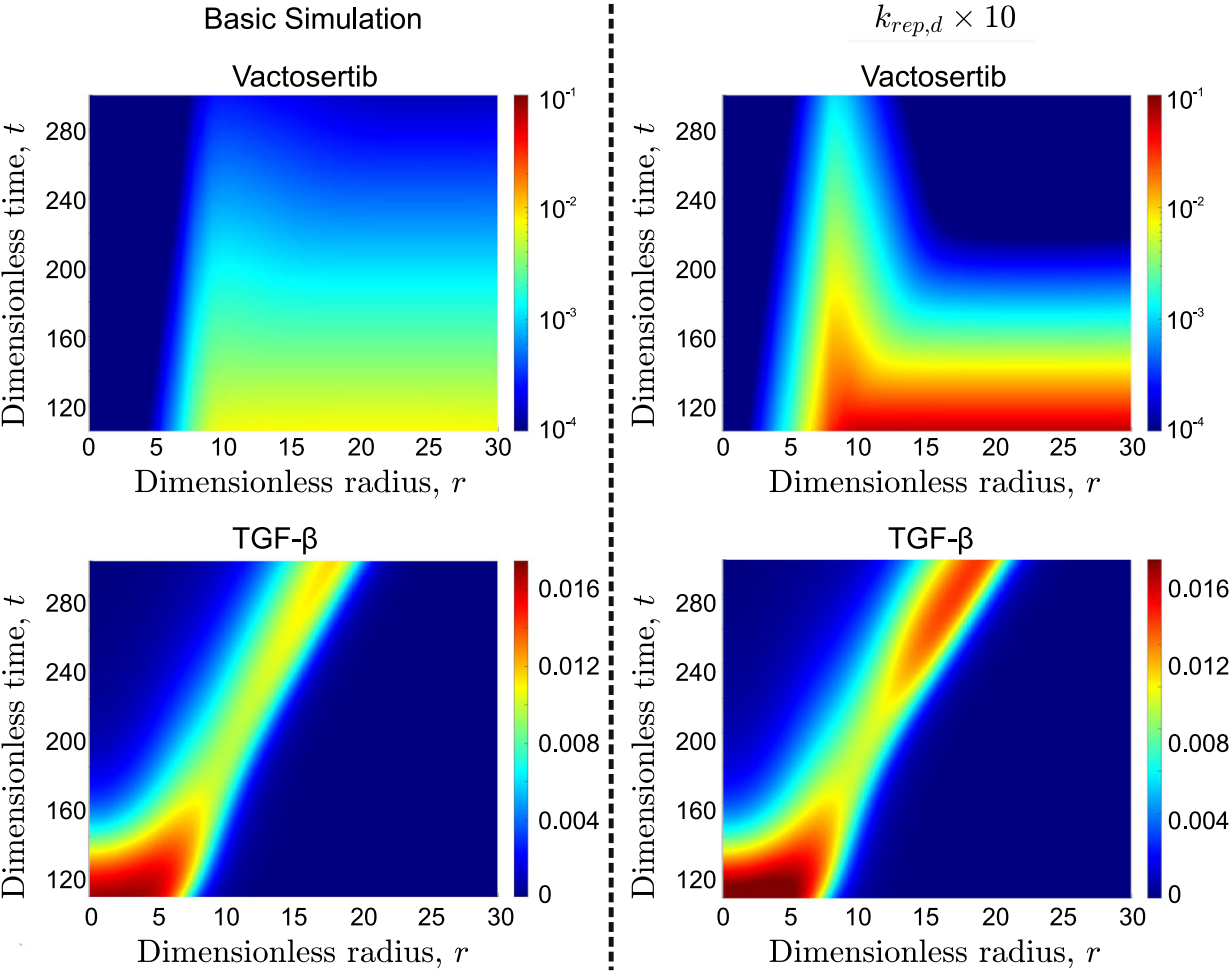
Evolution of averaged radial distributions of vactosertib (top row) and TGF-$\beta$ (bottom row) for the base case (left column) and $k_{rep,d}\times 10$ (right column)

Figure 16 (b) shows an interesting and unexpected result. Increasing the drug’s replenishment rate, $k_{rep,d}$, while initially offering a modest short-term benefit, ultimately leads to a more aggressive tumor characterized by an increased growth rate. This behavior can be better understood by comparing the spatial distributions of the drug and TGF-$\beta$.

Figure 17 shows the average radial distributions of vactosertib and TGF-$\beta$ for times $t\ge t_0^{im}$. Two cases are considered: the basic simulation and one where $k_{rep,d}$ is increased tenfold. Increasing $k_{rep,d}$ increases the influx of vactosertib into the system. $\hbox {M}_1$ macrophages present at that time, have their phenotype "frozen". This increase in $\hbox {M}_{1_p}$ macrophages explains the modest reduction in the tumor’s growth rate. With the drug’s concentration remaining high and the influx of macrophages insufficient to bind it, vactosertib diffuses within the tumor, gradually moving behind the advancing TGF-$\beta$ front formed by cancer cells, accumulating in an annulus extending from $r\approx 5$ to $r\approx 10$. These pockets of high drug concentration are further from new $\hbox {M}_1$ macrophages than TGF-$\beta$. Thus, as new macrophages penetrate the tumor, they encounter TGF-$\beta$ and adopt an $\hbox {M}_2$ phenotype before they encounter the drug. Such behaviors are not observed for the default $k_{rep,d}$. In the base-case, vactosertib is consumed by binding more gradually. Consequently, it diffuses less rapidly (due to the smaller spatial gradient) and does not accumulate behind the TGF-$\beta$ front. These results explain why if $k_{rep,d}$ values are large, immunotherapy may accelerate rather than slow down tumor growth.

## Conclusions

In this study, we simulate the growth of a tumor embedded within healthy tissue and its infiltration by macrophages. The model is based on mass and momentum balance equations applied to each cell phase (cellular species). To efficiently solve the model, we utilize the commercial software Comsol Multiphysics ^®^ and the Finite Elements Method (FEM).

The model captures a range of phenomena, particularly macrophage behavior, which is influenced by signals emitted by the tumor’s environment. To achieve this, the model takes into account chemical species closely related to the shaping of macrophage phenotype and activity. By representing each cellular population -healthy tissue, cancer cells, and macrophages- as interacting viscous phases, our model integrates chemotaxis, cell proliferation, interstitial fluid transport and mechanical cell-cell interactions within a unified framework. This formulation enables efficient simulation over large tissue domains, where agent-based models would be computationally infeasible. While discrete models provide finer resolution at the single-cell level, our continuum-level approach facilitates the study of emergent behaviors and spatial heterogeneity in cellular and cytokine distributions.

The present model effectively captures the diverging behaviors of macrophages, distinguishing the pro-tumor action of $\hbox {M}_2$ macrophages and the anti-cancer function of $\hbox {M}_1$ macrophages. Furthermore, the model correlates the $\hbox {M}_2$ to $\hbox {M}_1$ ratio with the aggressiveness of tumor expansion and consequently, with a poorer expected outcome for the patient. This correlation is supported by experimental observations (Jayasingam et al. 2020; Zhang et al. 2014). Moreover, the model provides valuable insights into the macrophage distributions within the tumor’s micro-environment. Specifically, it accurately depicts macrophage localization at the tumor’s periphery and the limited, yet noticeable, infiltration of macrophages into the tumor’s interior -a well documented behavior (Albert et al. 2017; Khurana et al. 2018). Moreover, a parametric study revealed the influence of certain parameters $\left( k_{aa},\,k_{ext,M_1},\,k_{c,M_1},\,k_{c,M_2}\right)$ on the produced dynamics.

The spatial distributions of macrophages are further explored through simulations of multi-seed originating tumors. These simulations showcase the formation of hypoxic niches capable of accommodating dense populations of pro-tumor macrophages. These results are consistent with clinical images (Albert et al. 2017; Khurana et al. 2018; Arlauckas et al. 2018). The simulation results show that in tumors originating from multi-seeds, $\hbox {M}_2$ macrophages thrive within the hypoxic pockets formed when two spheroids meet, whereas $\hbox {M}_1$ macrophages localize at the tumor periphery. This observation is consistent with experimental findings, in which TAMs found in hypoxic niches typically have the $\hbox {M}_2$ phenotype, and the localisation of TAMs in such intersections has been reported previously (Arlauckas et al. 2018). Moreover, the infiltration of these cells into hypoxic niches is associated with a poorer prognosis for patients (Vito et al. 2020).

The study of multi-seed originating tumors and the integration of immunotherapy at a later stage serve as a testament to the model’s versatility. Indeed, the presented model effectively accommodates the description of radically different physics and seamlessly integrates therapy without imposing a significant increase in computational demands. The inclusion of immunotherapy is based on the compound vactosertib, which has demonstrated the ability to prevent the binding of TGF-$\beta$ on macrophage receptors (Pubchem 2024; Malek et al. 2023) and is currently undergoing clinical trials as an anti-cancer treatment (Samsung Medical Center (Responsible Party (2024), Case Comprehensive Cancer Center (Responsible Party) (2024a, 2024b); Yonsei University (Responsible Party) (2024)). To the best of our knowledge, this is the first multiphase model to combine the study of macrophage behavior, the phenomenon of alternative activation, and the administration of TGF-$\beta$ receptor targeting immunotherapy.

The presented study offers ample opportunities for further research on tumor growth modeling. While the phenomenon of alternative activation is a central theme of the model, its counterpart, classical activation, remained unexplored. Investigating the conditions conducive to classical activation within the tumor’s environment presents a very intriguing topic with therapeutic implications. The present model serves as a natural foundation for a future comprehensive exploration of classical activation.

Additionally, in the present setting, only two populations of macrophages have been considered. Recent studies suggest (Eftimie 2020; Eftimie and Barelle 2021) the potential of consideration of multiple populations of macrophages and of continuous transitions between them. Similar approaches have been spearheaded by some of the present authors, e.g., in the context of the populations of stem cells (Celora et al. 2021, 2023), and represent natural extensions of the model presented herein.

An essential step in enhancing the translational relevance of our model is its quantitative validation using patient-specific data, including spatial macrophage distributions, vascular density maps, and cytokine profiles derived from imaging and histopathology (Arlauckas et al. 2018; Jain 2005). Once calibrated, the model could aid in the design of personalized immunotherapies by simulating how variations in dosing, timing, or drug mechanisms influence $\hbox {M}_1$/$\hbox {M}_2$ dynamics and tumor response. This framework could facilitate in silico clinical trials to inform treatment planning, predict resistance, and stratify patients based on immune-microenvironmental characteristics (Braman et al. 2022).

Regarding immunotherapy, the current model can accommodate other therapeutic factors. Macrophages represent an excellent target for a variety of immunotherapeutic compounds, several of which have undergone clinical trials (such as Lacnotuzumab [NCT02435680, NCT01643850] and Carlumab [NCT01204996]) (Duan and Luo 2021). To incorporate such therapies into the model, one can modify the production terms of relevant cytokines and macrophage subpopulations. For example, lacnotuzumab -a CSF-1/CSF-1R inhibitor- can be modeled by reducing the CSF-1 mediated recruitment and polarization of macrophages. It would be of particular interest to explore such combination therapies, since the mechanism considered herein based on vactosertib was not deemed sufficient (based on the mechanism considered herein) to lead to the full elimination of the cancer cell population. Moreover, the computational nature of the present study opens up the opportunity to alter the drug’s administration regimen. Although the biological feasibility of such a possibility merits further experimental studies, it is something we could explore computationally.

In future extensions of this work, incorporating vascular heterogeneity into the model would be particularly valuable, as it significantly influences macrophage infiltration and treatment response (Chu et al. 2024; Braman et al. 2022). Spatial variations in vessel density, permeability, and maturity can amplify heterogeneity in the spatial distributions of oxygen and cytokines, which in turn affect the localization and phenotype of infiltrating macrophages. For instance, poorly vascularized or dysfunctional regions may foster hypoxic niches that favor $\hbox {M}_2$ polarization, whereas well-perfused areas are more likely to support $\hbox {M}_1$ recruitment and effective drug delivery (Arlauckas et al. 2018; Vito et al. 2020). Incorporating these vascular features could provide deeper insights into the spatial organization of tumor-associated macrophages and enhance the model’s predictive capabilities, particularly in the context of immunomodulatory interventions.

Lastly, another category of immune cells that play a significant role in cancer biology is T-cells. The ability of T-cells to identify and destroy cancer cells has generated considerable scientific interest, and the development of agents to enhance their anti-tumor responses holds great promise (Ahmed et al. 2023). Such studies regarding the interplay of immune cells with other populations such as T-cells are presently of intense interest (see (Mohammad Mirzaei et al. 2023; Robertson-Tessi et al. 2012) for recent examples), rendering the current setting a natural playground for related future studies.

## Supplementary Information

Below is the link to the electronic supplementary material.

Supplementary file 1 (pdf 649 KB)

## Data Availability

The data that support the findings of this study are available from the corresponding author upon reasonable request.
